# The efficacy of acupuncture and related treatments on chronic pelvic inflammatory disease: a network meta-analysis

**DOI:** 10.3389/fmed.2025.1731543

**Published:** 2026-01-12

**Authors:** Yitong Zheng, Jintao He, Yijun Zheng, Dongyi Ni, Yong Wang

**Affiliations:** 1 The Affiliated TCM Hospital of Guangzhou Medical University, Guangzhou University of Chinese Medicine, Guangzhou, Guangdong, China; 2 Department of Gastroenterology, The Second Affiliated Hospital of Xi’an Jiaotong University, Xi’an, Shaanxi, China; 3 Medical College of Acupuncture Moxibustion and Rehabilitation, Guangzhou University of Chinese Medicine, Guangzhou, Guangdong, China

**Keywords:** acupuncture, non-acupuncture physical therapy, chronic pelvic inflammatory disease, network meta-analysis, treatment

## Abstract

**Objective:**

To compare the efficacy of different acupuncture and related therapies for chronic pelvic inflammatory disease (CPID) and their effects on inflammatory factors via Network Meta-Analysis.

**Methods:**

Eight databases were systematically searched (up to April 20, 2025) for randomized controlled trials (RCTs) of acupuncture-related therapies for CPID. Eighty-four RCTs involving 8,147 patients were included. Study quality was assessed using the Cochrane risk-of-bias tool, and data were analyzed with R software.

**Results:**

AI interventions showed higher total effectiveness than conventional treatment. SUCRA rankings for total effectiveness: moxibustion combined with warm needle (89.1%), acupoint application therapy combined with ultrasound drug penetration (88.0%), and acupuncture combined with cupping (86.8%) ranked highest, while conventional treatment was the lowest (0.2%). For inflammatory factors: acupuncture showed the best improvement in IL-2 (61.6%); acupoint injection was optimal for TNF-α regulation (95.8%); moxibustion combined with acupuncture best modulated IL-6 (92.6%); and acupoint injection was best for CRP regulation (99.4%).

**Conclusion:**

Acupuncture and related therapies are effective for CPID, with differences in regulating specific inflammatory factors, providing an evidence-based foundation. However, limitations like publication bias exist, warranting validation by high-quality studies.

**Systematic review registration:**

https://www.crd.york.ac.uk/prospero/, identifier CRD420250655000.

## 1 Introduction

Chronic Pelvic Inflammatory Disease (CPID) is a persistent inflammatory condition primarily affecting women of reproductive age, often resulting from inadequately treated acute episodes. It significantly impairs patients’ quality of life and is a major cause of infertility (1) and chronic pelvic pain (2), imposing a substantial burden on global healthcare systems due to its high prevalence and associated long-term complications. Antibiotic therapy remains the first-line clinical regimen (3); however, approximately 40% of patients experience poor outcomes due to antimicrobial resistance (4), hypersensitivity reactions (5), or treatment intolerance, leading to frequent recurrence and disease chronicity. This situation underscores the urgent need for effective complementary and alternative medicine (CAM) options to address these therapeutic challenges.

Acupuncture and related therapies within Traditional Chinese Medicine (TCM), such as moxibustion, acupoint injection, and warm acupuncture, have garnered increasing attention due to their multi-targeted anti-inflammatory mechanisms. Preclinical and clinical studies suggest potential pathways of action: electroacupuncture may reduce pro-inflammatory cytokine levels (e.g., TNF-α, IL-6) by modulating the NF-κ B signaling pathway (6); moxibustion at the Shen que acupoint has been shown to significantly improve reproductive outcomes (7); and acupoint injection therapy might alleviate autoimmune disease progression and modulate inflammatory cytokines (e.g., IL-10, IFN-γ) (8).

Despite these encouraging findings, significant limitations persist in the current evidence base. The relative efficacy ranking among nine commonly used acupuncture and related interventions remains unclear, and high-quality randomized controlled trials (RCTs) for direct comparisons are lacking (9). Furthermore, methods for assessing and reporting key inflammatory biomarkers (e.g., IL-2, TNF-α, IL-6, CRP) vary considerably across studies, hindering effective data integration and interpretation. Conventional pairwise meta-analysis methods are inadequate for fully utilizing the available indirect comparative evidence, preventing the construction of a comprehensive efficacy network encompassing all nine interventions.

To address these limitations, this study employs, for the first time, a Bayesian framework Network Meta-Analysis (NMA). We comprehensively compare these nine interventions, systematically and quantitatively assessing their efficacy in improving clinical symptoms (e.g., total effective rate) in CPID patients. Additionally, we analyze their modulatory effects on four key inflammatory biomarkers (IL-2, TNF-α, IL-6, CRP) to elucidate potential mechanistic differences. The findings of this study aim to provide high-level evidence to support individualized therapeutic decision-making for CPID and inform the optimal integration of acupuncture and related therapies within contemporary medical practice.

## 2 Data and methods

This study strictly adhered to the PRISMA (Preferred Reporting Items for Systematic Reviews and Meta-Analyses) reporting guidelines (10) to ensure transparency and reproducibility. The protocol was prospectively registered on the PROSPERO platform (11) (Registration number: CRD42025065500).

### 2.1 Literature search strategy

We systematically searched eight electronic databases for randomized controlled trials (RCTs) investigating various acupuncture modalities for chronic pelvic inflammatory disease (CPID): Embase, Cochrane Central Register of Controlled Trials (CENTRAL), Web of Science, PubMed, China National Knowledge Infrastructure (CNKI), Wan fang Data, and VIP Database (Database for Chinese Technical Periodicals), China biomedical literature service system (CBM). The search period spanned from database inception to April 20, 2025. To maximize comprehensiveness, we supplemented the electronic search by manually reviewing the reference lists of included studies. The search strategy employed a combination of subject headings (e.g., Me SH terms) and free-text keywords. Key English search terms included: “Pelvic Inflammatory Disease,” “Chronic Pelvic Inflammatory Disease,” “Acupuncture,” “Acupuncture Therapy,” “Electroacupuncture,” “Moxibustion,” “Acupoint Injection,” “Warm Needling,” “fire acupuncture,” “auricular acupuncture,” “electroacupuncture,” “Cuppin”,” “Randomized Controlled Trial,” and their relevant synonyms and variations (see Supplementary Table 1).

### 2.2 Inclusion criteria

Studies were included if they met the following criteria:

1. Study type: RCT.

(2) Participants: Female patients diagnosed with CPID (12), no age restriction.

(3) Interventions: Control group received conventional therapy, including drug treatment, routine care, etc. The treatment group received different acupuncture-related therapies, e.g., moxibustion, acupuncture, acupoint patch, floating needles, warm acupuncture, acupoint injection, cupping, either alone or combined with the control group.

### 2.3 Exclusion criteria

Studies were excluded based on the following:

(1)    Duplicate publications or literature with potentially overlapping data (i.e., same authors, data source, interventions, overlapping timelines).
(2)    Literature where only the abstract was available and full text was inaccessible.
(3)    Studies only comparing treatment duration without relevant outcomes.

### 2.4 Literature screening and data extraction

Two reviewers (Yijun Zheng and Jintao He) independently performed the literature screening and data extraction, respectively, according to the pre-defined criteria. Literature screening: Yijun Zheng screened the titles, abstracts, and subsequently the full texts of retrieved records to identify studies eligible for inclusion. Data extraction: Jintao He performed data extraction from the included studies using a standardized form. Extracted information included: (1) Basic information (first author, publication year); (2) Control and treatment interventions; (3) Specific intervention operation time and acupoints used; (4) Data on observed outcome indicators; (5) Basic participant information. Any discrepancies during screening or extraction were resolved through discussion or consultation with a third reviewer.

### 2.5 Risk of bias assessment

The methodological quality of the included RCTs was assessed independently by two reviewers using the Cochrane Risk of Bias tool (13). The assessment evaluated seven domains: random sequence generation, allocation concealment, blinding of participants and personnel, blinding of outcome assessment, incomplete outcome data, selective reporting, and other biases. Each domain was judged as “low risk,” “high risk,” or “some concerns” (referred to as “unclear” in some contexts). Risk of bias summary and graph were generated using Rev Man 5.3 software.

### 2.6 Statistical analysis

A multi-software analytical workflow was implemented in accordance with established standards for high-quality network meta-analyses (14). Risk of bias was assessed using Review Manager 5.4.1, and Bayesian network meta-analysis was conducted using Stata MP-64 16.0. Publication bias evaluation and result visualization were performed using the metafor and ggplot2 packages in R 4.4.3. For dichotomous outcomes such as efficacy rates, odds ratios (OR) and their natural logarithms (lnOR) were used as effect measures. For continuous outcomes, including inflammatory markers, either standardized mean differences (SMD) or mean differences (MD) were applied based on data standardization. Evidence networks were constructed and closed loops identified using network graphs. Heterogeneity was assessed with the *I*^2^ statistic, with a fixed-effects model applied when *I*^2^ ≤ 50% and a random-effects model otherwise. Inconsistency within closed loops was evaluated using node-splitting methods, and consistency or inconsistency models were selected based on *P*-values and 95% confidence intervals (CI). Model convergence was confirmed by iterative calculation of the potential scale reduction factor (PSRF), with values between 1.00 and 1.05 indicating satisfactory convergence. The surface under the cumulative ranking curve (SUCRA) was generated to rank interventions using the netrank command. Publication bias was assessed using adjusted funnel plots and Egger’s test. Finally, multiple outcome measures were integrated via network meta-analyses to establish a two-dimensional evaluation system incorporating both clinical relevance and statistical significance.

## 3 Results

### 3.1 Literature search results

**FIGURE 1:**
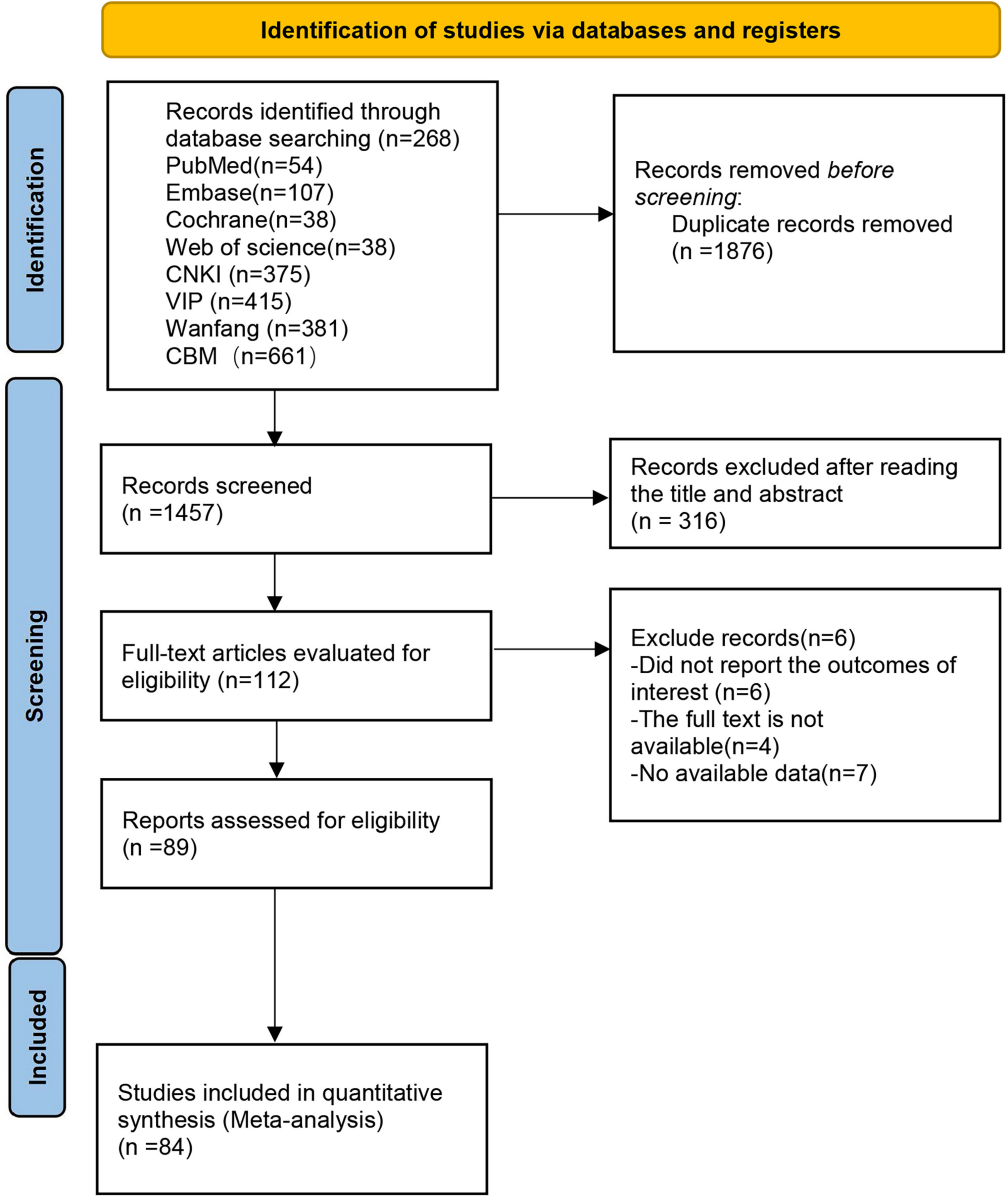
Flowchart for literature search and screening.

Initial screening identified 1,676 records. After removing duplicates and clearly irrelevant records, further screening based on study type, participants, interventions, and outcomes resulted in the inclusion of 84 articles (15–98). The screening process is detailed in Figure 1.

### 3.2 Basic characteristics of included literature after screening

A total of 84 RCTs involving 8,147 participants were included. Nine intervention types were involved: moxibustion (MOX), acupuncture (AC), traditional Chinese medicine (TCM), acupoint application therapy (AAT), ultrasonic drug penetration (UDD), floating needle (FN), warm needle moxibustion (WN), acupoint injection (AI), and cupping (CUP). Basic characteristics are summarized in Table 1.

**TABLE 1:** Basic characteristics of included RCTs.

Study	Year	Intervention	Sample size	Mean age	Course	Acupoint	Outcomes
M Liu (15)	2018	MOX_MTUT	30 30	32 31	once/15 min/d, once/15 min/d	−	F1, F2, F3
B Wang (16)	2024	MOX_ACUT	38 38	−−	once/30 min/donce/30 min/d	CV4, CV3, GV20	F1, F13
YH Kang (17)	2024	MOXUT	81 81	3130	once/30 min/d1 moxa cone 10 min, 2 once/d	CV6, ST29, ST36, SP6, BL	F1
YY He (18)	2024	MOX_ACAC	42 43	34 35	once/d, 30 min/once, once/d, 30 min/once	EX-CA1, CV4, ST29, CV3, BL32, SP6, ST36	F1
LH Wang (19)	2024	MOX_AATUT	35 35	36 36	once/d, 30 min/once, once/d, 6 h/once	CV8, CV4, CV6, ST36, SP6 EX-CA1, EX-CA1, CV6, CV3 GV4, BL23, BL28	F1, F3, F12, F13
YM Li (20)	2020	MOXUT	60 60	46 46	once/d, 20–30 min/once, once/d, 20–30 min/once	CV4, CV6, SP6, ST36, CV8	F2, F3
YP Wang (21)	2015	MOXUT	32 32	33 34	once/d, 30 min/once, once/d, 30 min/once	CV8, CV4, SP6, ST36	F1
XG Nong (22)	2017	MOXUT	42 42	34 34	once/d, 5–10 min/once, once/d, 5–10 min/once	CV4, CV6, SP6, ST36, CV8	F1, F3, F12, F13
MF Xu (23)	2015	MOXUT	46 46	–	once/d, 30 min/once, once/d, 30 min/once	CV8, CV6, CV4, CV3, EX-CA1	F1, F3, F13
XQ Yang (24)	2021	MOXUT	45 45	31 30	3 moxa cone/once, once/d3 moxa cone/once, once/d	CV4	F1, F12, F13
FH Cheng (25)	2023	AAT_UDDAAT	3434	58 58	once/d, 30 min/once, once/d, 30 min/once	EX-CA1, CV3, CV6, BL23, GV4, BL28, AP	F1, F3, F12, F13
HJ Wei (26)	2021	ACUT	53 53	37 36	once/d, 50 min/once, once/d, 50 min/once	–	F1, F2, F3, F13, F12
QY Pan (27)	2023	MOXUT	52 52	31 31	once/15 min/d, once/15 min/d	Fuke, HP	F1, F2, F3
DD Liu (28)	2023	FNUT	30 30	37 38	2 d/once, 2 min/once 2 d/once, 2 min/once	−*CPSTABLEENTER*−	F1, F13
GB Qi (29)	2018	AAT UT	9090	42 41	2 d/once 2 d/once	EX-CA1, CV6, ST25	F1, F13
SF Tian (30)	2022	AC UT	35 35	90 90	once/d, 20–30 min/once once/d, 20–30 min/once	BL	F1, F12
B Jiang (31)	2020	MOX_AC UT	30 30	38 39	once/d, 20∼30 min/once once/d, 20∼30 min/once	CV4, CV12	F1
Y Wu (32)	2021	FN UT	40 40	37 36	2 d/once, 2 min/once 2 d/once, 2 min/once	– –	F1, F13
L Luo (33)	2020	WN UT	50 50	34 33	once/d once/d	CV4, ST29, CV6, SP6	F1
XP Shi (34)	2017	AI UT	40 40	32 32	d/once d/once	CV4, EX-CA1	F3, F12, F13
Q Cheng (35)	2009	AI UT	107 107	20 46 20 46	−*CPSTABLEENTER*−	CV3, CV4, EX-CA1	F2, F3
Y Chen (36)	2017	MOX UT	125 125	42 42	d/once, 6–7 min/once d/once, 6–7 min/once	SP6, ST36, BL, ST39, EX-CA1, CV4 CV12	F2, F3
Y Li (37)	2016	MOX_ WN UT	132 130	- -	d/once, 30 min/once d/once, 30 min/once	−*CPSTABLEENTER*−	F1
C Zhang (38)	2022	AC UT	40 40	33 33	d/once, 30 min/once d/once, 30 min/once	CV6, CV4, CV3, SP6, ST36, EX-CA1	F1, F13
YC Huang (39)	2011	TCM_WN WN	55 50	31 2	d/once d/once	CV6, CV4, SP6, ST36 EX-CA1, BL23	F1
NN Kong (40)	2022	AAT UT	45 49	39 39	−*CPSTABLEENTER*−	CV6, EX-CA1, ST25	F1, F3, F13
HL Sang (41)	2017	AAT UT	90 90	31 32	8 h/once 8 h/once	CV8	F1, F3
L Shi (42)	2021	AC_WN WN	46 46	30 30	d/once, 30 min/once d/once, 30 min/once	BL23, CV4, GV4, SP6, BL32, SP10, BL18	F1, F3, F12
S Tao (43)	2016	MOX UT	30 30	36 38	d/once, 15–30 min/once d/once, 15–30 min/once	SP6, BL23, BL32	F1
J Liu (44)	2022	WN UT	30 30	36 36	2 d/once 2 d/once	ST25, CV6, CV4 EX-CA1, ST36, SP6	F1, F3, F12
X Jin (45)	2019	WN UT	40 40	32 32	d/once d/once	CV4, CV6, ST28, ST29, ST30, SP6	F1, F3, F12
L Wang (46)	2020	WN UT	60 60	31 32	d/once, 30 min/once d/once, 30 min/once	CV4, CV3, SP6	F1, F12
HX Xiao (47)	2024	AAT_WN UT	46 46	46 45	d/once, 30 min/once d/once, 30 min/once	CV4, ST36, CV6, EX-CA1, SP10, ST29	F1
XJ Xia (48)	2020	WN UT	45 45	35 34	d/once, 30 min/once d/once, 30 min/once	BL18, BL23, BL20, CV4, CV6, ST28, ST29	F1, F2, F3
L Shi a (49)	2021	WN UT	50 50	37 36	d/once, 30 min/once d/once, 30 min/once	BL18, BL23, BL20, CV4, CV6, SP6, ST36	F1, F12, F13
WX Tang (50)	2021	WN UT	55 55	34 35	d/once, 30 min/once d/once, 30 min/once	SP10 CV4 CV3 ST36 SP6 EX-CA1 ST29	F1, F2, F3
JR Zhou (51)	2015	WN_TCM WN	30 30	38 38	d/once d/once	CV4, CV6, EX-CA1, SP10, SP6, KI3, LR3, SP9, BL23, BL32, BL52	F1, F13
HL Zheng (52)	2008	WN UT	45 40	33 34	d/once, 40 min/once d/once, 40 min/once	CV4, CV6, ST36, BL23	F1, F13
J Wang (53)	2023	WN UT	43 43	36 36	once/2 d, 30 min/once once/2 d, 30 min/once	BL23, BL20, BL18, ST36, SP6, CV4, CV6	F1, F12
LJ Chen (54)	2019	AAT UT	40 40	37 35	once/2 d, 30 min/once once/2 d, 30 min/once	CV3, CV4, CV6, CV8, EX-CA1, BL32	F1
YM Yu (55)	2023	AAT UT	70 70	36 36	once/d, 2–4 h/once once/d, 2–4 h/once	CV4, CV6, EX-CA1, CV3	F1, F3, F13
XJ Song (56)	2022	AAT UT	50 50	36 57	2 d/once, 4 h/once 2 d/once, 4 h/once	CV4, CV6, EX-CA1, CV3	F1, F3, F13
SY Huang (57)	2018	AI UT	50 50	32 32	once/d once/d	CV4, CV3, EX-CA1, EX-LE3	F12
W Hu (58)	2023	MOX_AC UT	40 40	44 43	d/once, 30 min/once d/once, 30 min/once	CV12, CV10, CV6, CV4	F1, F3, F12, F13
GR Shi (59)	2021	AC UT	45 45	32 33	d/once, 30 min/once d/once, 30 min/once	CV3, BL31-34, CV6, CV4, ST25, CV12, CV17, GV20	F1, F3, F13
Y Yuan (60)	2024	WN UT	34 34	45 45	−*CPSTABLEENTER*−	ST36, CV4, CV3, EX-CA1, BL23, GV4, EX-BL7, SP6	F1, F2, F3, F13
LH Qiu (61)	2019	AC_MOX_CUP UT	55 52	42 41	d/once, 30 min/once d/once, 30 min/once	ST36, BL23, BL54, ST29, CV3, EX-CA1, SP6, CV4, BL32	F1
QC Yi (62)	2018	MOX_AC AC	45 45	29 29	d/once d/once	CV4, AX-CA1, BL25, BL32, SP6	F1, F13
P Zhou (63)	2014	AC_TCM UT	80 40	29 29	d/once, 30 min/once d/once, 30 min/once	SP6, CV6, CV4, ST29, SP10, LR2, SP9	F1
Y Tian (64)	2022	AAT UT	35 35	38 38	2 d/once 2 d/once	EX-CA1, ST25, CV6	F13
MX Liao (65)	2014	AC UT	30 30	34 34	once/d, 30 min/once once/d, 30 min/once	ST25, CV4, CV3, EX-CA1, SP10, SP6, ST28, SP9, LI4, LR3, SP8, CV6, ST36	F1, F12
MX Liao a (66)	2014	AC UT	35 35	33 34	once/d, 30 min/once once/d, 30 min/once	CV3, EX - CA1, SP10, SP6, ST25, CV4	F1, F13
BK Li (67)	2016	AC UT	30 30	33 33	once/d, 30 min/once once/d, 30 min/once	SP6, ST25, SP10, LI4, ST28, SP8, ST36, SP9	F1, F12
XQ Huang (68)	2023	AC UT	61 61	41 42	once/d, 20 min/once once/d, 20 min/once	CV4, CV6, CV8, SP6	F1, F3, F12, F13
ZX Cheng (69)	2020	AC UT	30 30	41 35	once/d, 40 min/once once/d, 40 min/once	DU20, CV4, CV6, ST36, SP6, ST29, EX - CA1, CV3, LR3	F1
J Li (70)	2021	MOX UT	45 45	36 34	once/d, 30 min/once once/d, 30 min/once	CV3, CV6, ST36, GB34, SP6, SP10	F1
T Nie (71)	2023	AC UT	41 41	34 35	once/d, 40 min/once once/d, 40 min/once	CV3, CV4, CV6, ST25, SP6, ST36	F1, F13
GL Shi (72)	2017	AC UT	43 43	38 38	once/d, 30 min/once once/d, 30 min/once	CV3, EX - CA1, SP10, SP6, ST25, CV4	F1, F13
LY Liu (73)	2010	AC UT	59 59	35 34	once/d, 30 min/once once/d, 30 min/once	CV6, CV3, SP10, ST36, SP9, SP6	F1
HX Tao (74)	2009	AC UT	30 30	35 34	once/d, 30 min/once once/d, 30 min/once	CV6, CV3, SP10, ST36, SP9, SP6	F1
J Lu (75)	2013	AC UT	52 51	– –	once/d, 30 min/once once/d, 30 min/once	ST25, CV4, EX - CA, SP6	F1
L Tian (76)	2020	AC UT	72 72	36 36	once/d, 30 min/once once/d, 30 min/once	CV4, ST28, ST29, BL23, BL32	F1, F2, F3, F13
F Liao (77)	2019	AC_CUP UT	60 60	45 46	once/d, 30 min/once once/d, 30 min/once	CV4, CV3, EX - CA1, ST29, SP6, ST36, BL23	F1, F3, F12, F13
XL Huang (78)	2018	AC_AI UT	40 40	32 32	once/d, 30 min/once once/d, 30 min/once	CV6, CV3, SP6, SP9, ST36	F1, F2, F3
LH Li (79)	2021	AC UT	80 80	38 39	once/d, 30 min/once once/d, 30 min/once	BL32, CV4, SP6, CV6, BL31, BL26	F1, F13
CX Yang (80)	2024	AC UT	43 43	31 32	once/d, 30 min/once once/d, 30 min/once	CV4, BL23, BL32, ST28, ST29, CV6, SP6, SP10, EX - CA1	F1
RR Cheng (81)	2023	AC UT	30 30	34 34	once/d, 30 min/once once/d, 30 min/once	ST36, SP6, EX - CA1, CV6, CV3	F1, F3, F12
JJ Cheng (82)	2017	AC UT	60 60	36 34	once/d, 30 min/once once/d, 30 min/once	CV4, CV3, SP10, ST36, SP6, SP9, ST29, EX - CA1	F1, F3, F12
J Mo (83)	2019	AC UT	40 40	36 36	once/d, 30 min/once once/d, 30 min/once	EX - CA1, ST29, SP10, CV4, CV3, SP6, GB34, ST36	F1
X Tian (84)	2018	AC UT:AC_CUP	30 30	34 34	once/d, 30 min/once once/d, 30 min/once	SP6, CV4, ST29, CV3, EX - CA1, BL32, ST36, BL23, BL54	F1
X Liu (85)	2020	AC UT	50 50	36 34	once/d, 30 min/once once/d, 30 min/once	CV4, ST29, BL32, CV3, EX - CA1, ST36, SP6, BL23, BL54	F1
H Yan (86)	2008	AC UT	30 30	45 45	once/d, 30 min/once once/d, 30 min/once	BL23, BL32, BL37, SP6	F1
R Shi (87)	2021	AC UT	35 35	37 37	once/d, 30 min/once once/d, 30 min/once	ST36, GV4, EX - BL7, CV4, BL23, EX - CA1	F1, F3, F12, F13
LZ Chen (88)	2014	AC UT	38 38	33 33	once/d, 30 min/once once/d, 30 min/once	CV6, CV3, CV4, EX - CA1, SP6, ST36, BL20, BL30, BL32, BL23, BL25	F1
C Zhang (89)	2024	AC UT	30 30	32 32	once/d, 30 min/once once/d, 30 min/once	CV6, CV3, CV4, EX - CA1, ST36, SP6	F1, F3, F12, F13
WC Li (90)	2014	AC UT	50 50	35 34	once/d, 30 min/once once/d, 30 min/once	ST36, CV3, PC6, BL25, SP6, CV4, ST29, GB34, SP9	F1, F2, F3
XL Luo (91)	2022	AC UT	51 51	32 32	once/d, 30 min/once once/d, 30 min/once	CV8, CV4, BL23, BL20	F1
XQ Liu (92)	2023	AC UT	30 30	38 37	once/d, 30 min/once once/d, 30 min/once	GV3, SP6	F2, F3, F13
MP Dai (93)	2015	MOX UT	84 81	31 32	once/d, 30 min/once once/d, 30 min/once	CV3, EX-CA1, ST36, SP6	F1
DB Wu (94)	2015	AAT UT	50 50	32 34	Once/4–6 h, 24 h/once once/4–6 h, 24 h/once	GB26, CV6, SP6, CV3, SP8, BL32	F1, F2, F13
YE Wu (95)	2023	AAT UT	43 43	42 42	once/d, 6 h/once once/d, 6 h/once	SP6, GB26, CV3	F3, F12, F13
ZY Ye (96)	2023	AC UT	43 43	41 41	once/d, 30 min/once once/d, 30 min/once	GB26, EX-CA1, SP10, SP6, CV3, SP9, CV4, ST36, BL23	F1
YX Miao (97)	2015	MOX_AC UT:AC	50 50	28 28	once/d, 30 min/once once/d, 30 min/once	SP6, ST29, SP10, CV6, LR2, CV4	F1
HL Zhen (98)	2008	WN TCM	45 40	33 34	once/d 40 min/once once/d 40 min/once	CV4, CV6, ST36, BL23	F1, F13

MOX, moxibustion; AC, acupuncture; TCM, traditional Chinese medicine; AAT, Acupoint application therapy; UDD, ultrasonic drug penetration FN, floating needle; WN, warm needle moxibustion; AI, acupoint injection; CUP, cupping; UT, conventional treatment. F1, curative effect; F2, IL-2; F3, TNF-a; F12, IL-6; F13, CRP. “–” Means: not report.

### 3.3 Inclusion of study risk of bias assessment

The methodological quality of the 84 included studies was evaluated using the Cochrane Risk of Bias tool, assessing seven domains: random sequence generation, allocation concealment, blinding of participants and personnel, blinding of outcome assessment, incomplete outcome data, selective reporting, and other biases. The assessment results for each domain are detailed below:

1. Random Sequence Generation: Low Risk: 49 studies (59.8%) employed standardized methods (e.g., random number tables, computer randomization). Unclear Risk: 19 studies (23.2%) provided insufficient detail regarding the randomization procedure. High Risk: 9 studies (11.0%) utilized potentially biased sequence generation methods, introducing risk of selection bias.
2. Allocation Concealment: Low Risk: 27 studies (32.9%) implemented adequate concealment methods (e.g., sealed envelopes, central randomization). Unclear Risk: 25 studies (30.5%) did not report sufficient information to judge concealment. High Risk: 32 studies (39.0%) failed to conceal allocation adequately, allowing researcher access to group assignments prior to participant enrollment and substantially elevating selection bias risk.
3. Blinding of Participants and Personnel: Low Risk: 30 studies (36.6%) employed strategies to blind participants and/or personnel (e.g., placebo, sham procedures, blinding training), minimizing performance bias. Unclear Risk: 8 studies (9.8%) provided inadequate information regarding blinding implementation. High Risk: 39 studies (47.6%) explicitly did not blind participants and personnel due to the nature of the intervention, potentially influencing the measurement of intervention effects.
4. Blinding of Outcome Assessment: Low Risk: 28 studies (34.1%) implemented methods to blind outcome assessors (e.g., independent assessment, blinded adjudication), reducing detection bias risk. Unclear Risk: 6 studies (7.3%) lacked clear reporting on outcome assessor blinding. High Risk:43 studies (52.4%) did not blind outcome assessors, introducing potential detection bias if assessors were aware of group assignments.
5. Incomplete Outcome Data: Low Risk:65 studies (79.3%) appropriately handled missing outcome data (e.g., intention-to-treat analysis). Unclear Risk:10 studies (12.2%) failed to report attrition/exclusion rates or reasons. High Risk: 2 studies (2.4%) exhibited high risk due to substantial missing data or inappropriate handling (e.g., high loss-to-follow-up rates, per-protocol analysis with significant exclusions).
6. Selective Reporting: Low Risk: 72 studies (87.8%) reported outcomes consistent with pre-specified protocols or trial registries. Unclear Risk: 6 studies (7.3%) lacked accessible protocols or registrations, precluding definitive assessment. High Risk: No studies (0%) were judged as high risk for selective outcome reporting. Overall reporting completeness was good.
7. Other Potential Sources of Bias: Low Risk: 14 studies (17.1%) addressed potential confounding biases (e.g., disclosed funding sources, reported balanced baseline characteristics). Unclear Risk:64 studies (78.0%) did not report sufficient information on potential biases such as funding conflicts, baseline imbalances, or other study-specific limitations. High Risk: No studies (0%) were definitively judged as high risk based on reported information. The potential impact of unreported biases requires consideration, potentially through subgroup analyses.

**FIGURE 2:**
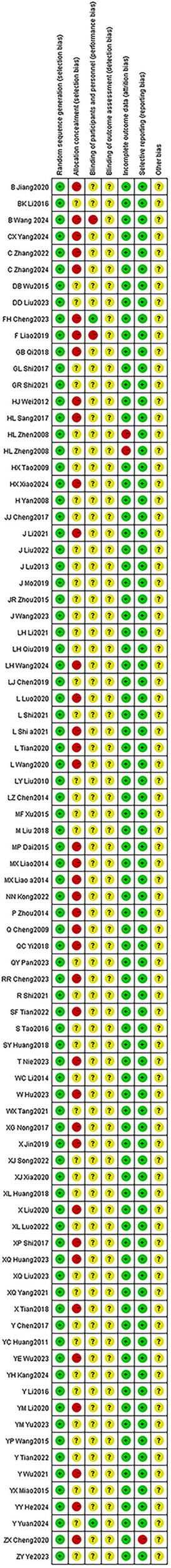
Bias risk ratio chart.

**FIGURE 3:**
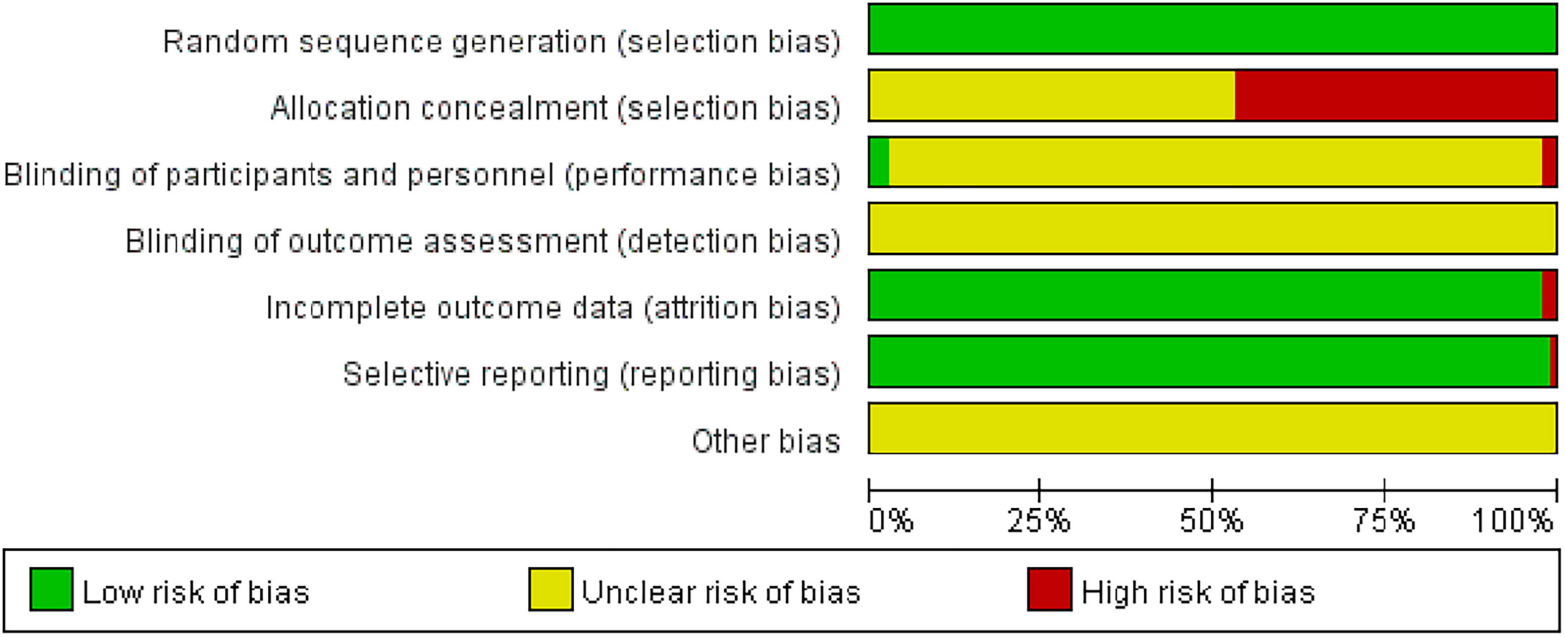
Bias risk summary chart.

Among the assessed domains, selective reporting exhibited the lowest overall risk of bias. However, significant methodological concerns were identified: Allocation concealment posed a high risk in a substantial proportion of studies (39.0%, *n* = 32). Deficiencies in random sequence generation introduced high risk in 11.0% (*n* = 9) of studies. Inadequate blinding of participants/personnel (high risk: 47.6%, *n* = 39) and blinding of outcome assessment (high risk: 52.4%, *n* = 43), along with unclear reporting on blinding strategies, were prevalent issues. Specific high-risk judgments for blinding implementation occurred in 8 studies for participants/personnel and 6 were unclear for outcome assessment. These limitations, particularly in selection bias (randomization, concealment) and performance/detection bias (blinding), may compromise the stability and validity of pooled effect estimates. Subsequent sensitivity analyses are warranted to explore the potential influence of these biases. These methodological limitations must be thoroughly considered when interpreting the overall findings of this systematic review. Graphical representations of the risk of bias assessments across all included studies and domains are provided in Figures 2, 3.

### 3.4 Direct comparison results

Prior to the network meta-analysis, a direct comparison meta-analysis was performed on the 84 included RCTs to evaluate pairwise intervention effects. Results are summarized in Table 2. Heterogeneity was assessed using the I^2^ statistic: a fixed-effect model was applied when I^2^ ≤ 50%, and a random-effects model when I^2^ > 50%. For dichotomous outcomes (e.g., overall effectiveness), odds ratios (OR) with 95% confidence intervals (CI) were used; for continuous outcomes (e.g., inflammatory factors), mean differences (MD) with 95% CI were used.

**TABLE 2:** Direct comparison heterogeneity test results table.

Outcomes	Pairwise meta-analysis	No of study	Heterogeneity (%)	OR 95%CrI
F1 (Effectiveness)	AAT_UDD VS. AAT	1	NA	16.25 (1.88, 999.38)
	UT vs. AAT	7	0	0.21 (0.12, 0.37)
	AC_CUP vs. AC	1	NA	9.88 (0.81, 173.81)
	MOX_AC vs. AC	5	0	6.04 (2.51, 16.10)
	UT vs. AC	28	0	0.20 (0.05, 0.81)
	UT vs. AC_AI	2	0	0.17 (0.02, 0.88)
	UT vs. MOX	9	60.60	0.32 (0.10, 0.96)
	WN vs. UT	8	0	14.28 (0.98, 5.96)
	WN vs. TCM_WN	2	0	0.27 (0.10, 0.73)
**Outcomes**	**Pairwise meta-analysis**	**No of study**	**Heterogeneity (%)**	**MD 95%CrI**
F2(IL-2)	UT vs. AAT	1	NA	−1.89 (−11.94, 8.20)
	UT vs. AC	5	98.80	−0.24 (−5.28, 4.78)
	UT vs. AC_AI	1	NA	−0.60 (−1.09, 9.47)
	UT vs. Al	2	99.60	−8.86 (−17.56, −1.73)
	UT vs. MOX	4	99.30	−0.48 (−5.48, 4.53)
	WN vs. UT	3	99.70	0.75 (−5.26, 6.74)
**Outcomes**	**Pairwise meta-analysis**	**No of study**	**Heterogeneity (%)**	**MD 95%CrI**
F3(TNF-α)	AAT_UDD vs. AAT	1	NA	−12.39 (−37.99, 12.90)
	UT vs. AAT	6	99.40	10.30 (−1.00, 21.65)
	UT vs. AC	10	100	5.06 (−2.92, 13.00)
	UT vs. AC_AI	1	NA	0.57 (−24.78, 25.84)
	UT vs. AC_CUP	1	NA	0.45 (−24.77, 25.68)
	WN vs. AC_WN	1	NA	0.77 (−24.47, 25.80)
	UT vs. Al	2	100	32.30 (6.43, 50.24)
	UT vs. MOX	5	100	6.55 (−4.66, 17.87)
	UT vs. MOX_AAT	1	NA	0.50 (−24.60, 25.81)
	UT vs. MOX_AC	1	NA	0.81 (−24.32, 26.26)
	WN vs. UT	5	100	5.55 (-16.83, 5.83)
**Outcomes**	**Pairwise meta-analysis**	**No of study**	**Heterogeneity (%)**	**MD 95%CrI**
F12(IL-6)	AAT_UDD vs. AAT	1	NA	−1.76 (−152.57, 149.07)
	UT vs. AAT	1	NA	4.08 (−145.99, 154.14)
	UT vs. AC	10	100	20.73 (−29.62, 70.84)
	UT vs. AC_CUP	1	NA	51.95 (−99.65, 201.95)
	WN vs. AC_WN	1	NA	11.05 (−138.43, 161.64)
	UT vs. Al	2	99.90	−72.06 (−184.05, 34.86)
	UT vs. MOX	2	99.80	103.44 (−5.47, 214.22)
	UT vs. MOX_AAT	1	NA	0.34 (−150.24, 151.38)
	UT vs. MOX_AC	1	NA	179.58 (20.34, 338.96)
	WN vs. UT	5	100	−190.73 (-205.47, −175.99)
**Outcomes**	**Pairwise meta-analysis**	**No of study**	**Heterogeneity (%)**	**MD 95%CrI**
F13	AAT_UDD vs. AAT	1	NA	−1.76 (−152.57, 149.07)
	UT vs. AAT	1	NA	4.08 (−145.99, 154.14)
	UT vs. AC	10	100	20.73 (−29.62, 70.84)
**Outcomes**	**Pairwise meta-analysis**	**No of study**	**Heterogeneity (%)**	**MD 95%CrI**
F1 (Effectiveness)	AAT_UDD VS. AAT	1	NA	16.25 (1.88, 999.38)
	UT vs. AC_CUP	1	NA	51.95 (−99.65, 201.95)
	WN vs. AC_WN	1	NA	11.05 (−138.43, 161.64)
	UT vs. Al	2	99.9	−72.66 (−184.05, 34.88)
	UT vs. MOX	2	99.8	103.44 (−5.47, 214.22)
	UT vs. MOX_AAT	1	NA	0.34 (−150.24, 151.38)
	UT vs. MOX_AC	1	NA	179.58 (20.34, 338.96)
	WN vs. UT	5	100	−190.73 (−205.47, -175.99)

#### 3.4.1 F1 (effectiveness)

Nine direct comparisons were analyzed. AAT_UDD showed superior effectiveness over AAT (OR = 16.25, 95% CI: 1.88–999.38). Both AC_CUP (OR = 9.88, 95% CI: 0.81–173.81) and MOX_AC (OR = 6.04, 95% CI: 2.51–16.10) were more effective than AC alone. Multiple therapies—including WN, AAT, AC, AC_AI, and MOX—demonstrated higher effectiveness than UT (all OR < 0.37, *I*^2^ = 0–60.6%). TCM_WN also outperformed WN alone (OR = 0.27, 95% CI: 0.10–0.73).

#### 3.4.2 F2 (IL-2)

Among six comparisons, only AI significantly reduced IL-2 levels compared to UT (MD = –8.86, 95% CI: −17.56 to −1.73), despite high heterogeneity (*I*^2^ = 99.6%). All other comparisons showed no significant differences with wide confidence intervals.

#### 3.4.3 F3 (TNF-α)

AI was superior to UT in reducing TNF-α (MD = 32.30, 95% CI: 6.43–50.24), though heterogeneity was high (*I*^2^ = 100%). None of the other ten comparisons showed statistically significant differences.

#### 3.4.4 F12 (IL-6)

MOX_AC significantly lowered IL-6 compared to UT (MD = 179.58, 95% CI: 20.34–338.96). No other comparisons among the ten analyzed reached statistical significance, with several showing high heterogeneity (*I*^2^ up to 100%).

#### 3.4.5 F13 (CRP)

None of the ten direct comparisons for CRP showed statistically significant differences. High heterogeneity was observed in key comparisons such as UT vs. AI (*I*^2^ = 99.9%) and UT vs. MOX (*I*^2^ = 99.8%).

### 3.5 Evidence map of reticulation

**FIGURE 4:**
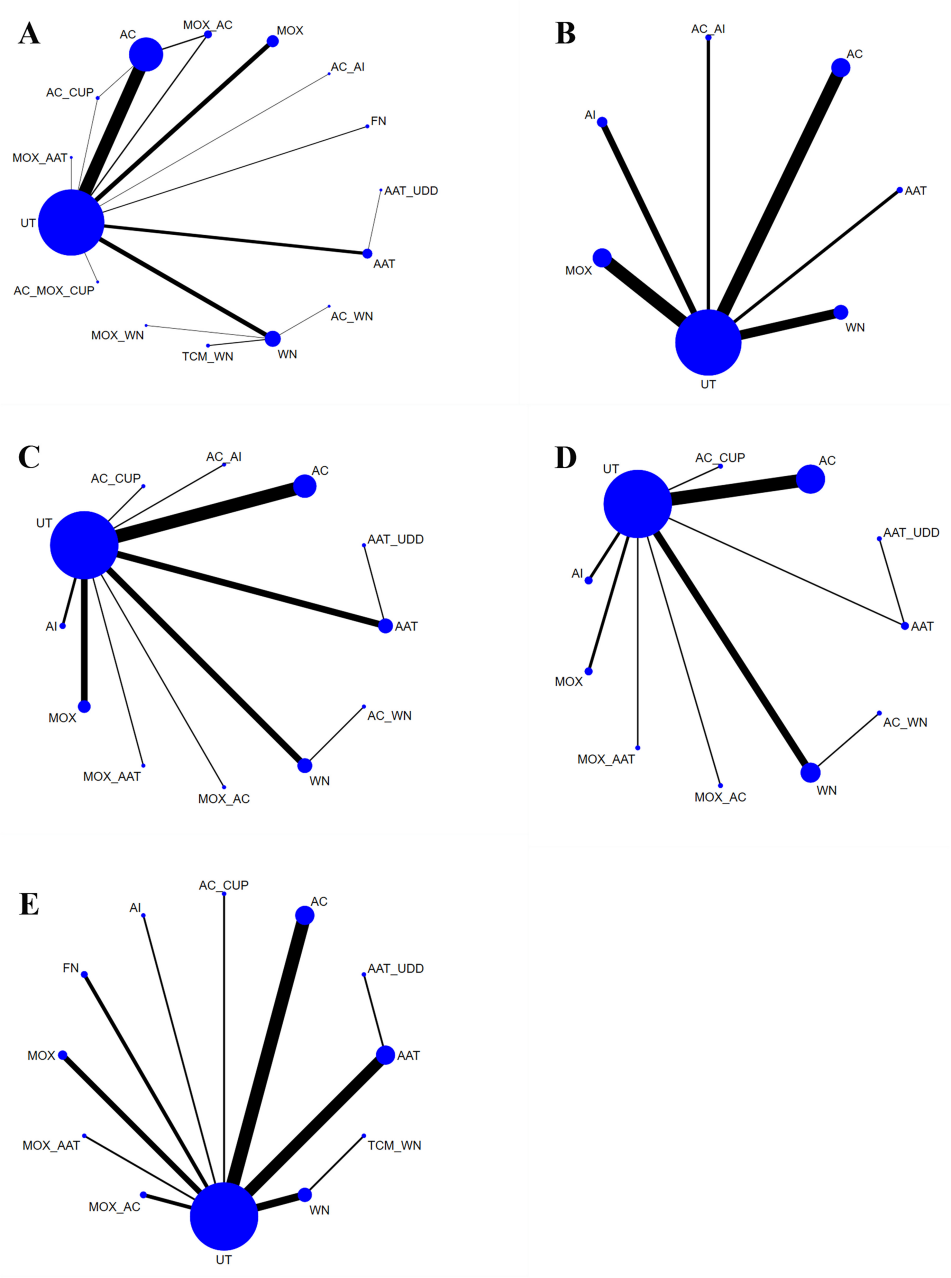
Network plot of meta-analysis. **(A)** Efficacy. **(B)** IL-2. **(C)** TNF-α. **(D)** IL-6. **(E)** CRP.

F1 (Effectiveness): 73 papers covered 15 interventions. Frequent comparisons existed between AAT_UDD, MOX_WN, and AC_MOX_CUP. Three closed loops were present: Loop 1: AAT_UDD—MOX_WN—AC_MOX_CUP; Loop 2: MOX_WN—TCM_WN—WN; Loop 3: AC_MOX_CUP—WN—MOX_WN (see Figure 4A).

F2 (IL-2): 15 papers covered 7 interventions. Frequent comparisons between AC and AI. One closed loop: AC—AI—MOX—AC (see Figure 4B).

F3 (TNF-α): 33 papers covered 12 interventions. Frequent comparisons between AC and AI. One closed loop: AC—AI—MOX—AC (see Figure 4C).

F12 (IL-6): 24 papers covered 11 interventions. Frequent comparisons between MOX_AAT and AAT_UDD (see Figure 4D).

F13 (CRP): 29 papers covered 12 interventions. Frequent comparisons between MOX_AAT and AI. One closed loop: AI—MOX_AAT—AC_CUP—AI (see Figure 4E).

### 3.6 Results of reticulated meta-analysis under the consistency model and cumulative probability ranking

Under the consistency model, all PSRF values equaled 1.00, indicating good model stability. Results comparing different interventions against acupuncture (AC) and conventional treatment (CT, UT) are presented below (Tables 3, 4 and Supplementary Tables 2–5 summarize pairwise comparisons).

**TABLE 3:** Results of network meta-analysis of efficacy.

OR95%Cri														
AAT														
0.07 (0, 0.5)*	AAT_UDD													
0.91 (0.51, 1.67)	13.08 (1.65, 383.89)*	AC												
0.82 (0.1, 4.25)	11.97 (0.64, 465.13)	0.91 (0.11, 4.36)	AC_AI											
0.08 (0, 0.6)*	1.13 (0.02, 52.4)	0.09 (0, 0.61)*	0.1 (0, 1.67)	AC_CUP										
1.12 (0.26, 4.07)	16.32 (1.35, 546.93)*	1.23 (0.3, 4.07)	1.37 (0.17, 14.3)	14.37 (1.26, 517.84)*	AC_MOX_ CUP									
0.23 (0.04, 1.03)	3.44 (0.24, 126.88)	0.26 (0.05, 1.06)	0.29 (0.03, 3.45)	3.02 (0.22, 114.68)	0.21 (0.03, 1.45)	AC_WN								
0.49 (0.07, 2.26)	7.07 (0.43, 268.88)	0.54 (0.08, 2.28)	0.59 (0.05, 7.53)	6.31 (0.38, 251.44)	0.44 (0.05, 3.14)	2.09 (0.19, 18.64)	FN							
1.51 (0.79, 2.96)	21.82 (2.69, 644.24)*	1.67 (1.02, 2.69)*	1.83 (0.37, 15.63)	19.22 (2.59, 595.72)*	1.36 (0.39, 5.62)	6.45 (1.53, 35.49)*	3.07 (0.71, 22.19)	MOX						
0.8 (0.09, 4.21)	11.64 (0.62, 467.49)	0.88 (0.11, 4.32)	0.97 (0.07, 13.18)	10.31 (0.57, 411.35)	0.71 (0.07, 5.79)	3.4 (0.29, 33.6)	1.63 (0.13, 18.94)	0.53 (0.06, 2.66)	MOX_AAT					
0.23 (0.09, 0.57)*	3.37 (0.37, 102.84)	0.26 (0.12, 0.51)*	0.28 (0.05, 2.62)	2.98 (0.35, 94.45)	0.21 (0.05, 0.97)*	0.99 (0.2, 6.07)	0.47 (0.09, 3.66)	0.15 (0.06, 0.35)	0.29 (0.05, 2.6)	MOX_AC				
0.07 (0, 0.49)*	0.94 (0.02, 44.32)	0.07 (0, 0.51)*	0.08 (0, 1.43)	0.83 (0.02, 41.64)	0.06 (0, 0.63)**	0.28 (0.01, 3.41)	0.13 (0, 2.08)	0.04 (0, 0.31)	0.08 (0, 1.39)	0.29 (0.01, 2.28)	MOX_WN			
0.28 (0.08, 0.88)*	4.1 (0.39, 133.39)	0.31 (0.1, 0.87)*	0.34 (0.05, 3.45)	3.64 (0.36, 121.35)	0.25 (0.05, 1.37)	1.2 (0.23, 7.58)	0.58 (0.09, 4.95)	0.19 (0.06, 0.55)*	0.35 (0.05, 3.46)	1.21 (0.32, 4.3)	4.26 (0.5, 141.73)	TCM_WN		
4.53 (2.74, 7.82)*	65.38 (8.43, 1895.5)*	5 (3.81, 6.61)*	5.49 (1.18, 45.4)*	57.48 (8.11, 1764.02)*	4.07 (1.27, 15.98)*	19.34 (4.84, 102.68)*	9.19 (2.25, 63.93)*	3 (2.03, 4.51)*	5.68 (1.19, 45.33)*	19.47 (9.53, 43.04)*	68.03 (10.08, 2156)*	16.15 (5.95, 48.54)*	UT	
1.02 (0.52, 2.03)	14.8 (1.81, 439.77)*	1.13 (0.67, 1.87)	1.24 (0.25, 10.58)	13.03 (1.72, 407.2)*	0.92 (0.26, 3.82)	4.34 (1.17, 21.94)*	2.08 (0.47, 15.1)	0.68 (0.37, 1.21)	1.28 (0.25, 10.63)	4.4 (1.89, 10.81)*	15.18 (2.4, 472.14)*	3.63 (1.47, 9.99)*	0.23 (0.14, 0.34)*	WN

*(*P* < 0.05) valid.

**TABLE 4:** Results of network meta-analysis of IL-2.

MD95% Cri						
AAT						
1.67 (−9.59, 12.96)	AC					
1.31 (−12.88, 15.57)	−0.34 (−11.55, 10.94)	AC_AI				
−6.92 (−20.75, 4.96)	−8.6 (−18.96, −0.06)	−8.23 (−22.03, 3.74)	AI			
1.43 (−9.82, 12.65)	−0.24 (−7.3, 6.86)	0.11 (−11.12, 11.4)	8.38 (−0.18, 18.75)	MOX		
1.9 (−8.18, 11.98)	0.24 (−4.77, 5.24)	0.61 (−9.45, 10.65)	8.86 (1.68, 17.67)*	0.48 (−4.55, 5.49)	UT	
1.13 (−10.57, 12.9)	−0.53 (−8.34, 7.35)	−0.16 (−11.89, 11.52)	8.08 (−1.06, 18.96)	−0.3 (−8.09, 7.5)	−0.77 (−6.8, 5.26)	WN

*(*P* < 0.05) valid.

#### 3.6.1 F1 (effectiveness)

Compared to CT (UT), patients with CPID showed significantly increased effectiveness after treatment with: Acupoint application therapy (AAT vs. UT: OR = 4.53, 95%CrI = 2.74–7.82); Acupoint application therapy + ultrasonic drug penetration (AAT_UDD vs. UT: OR = 65.38, 95%CrI = 8.43–1895.5); Acupuncture + acupoint injection (AC_AI vs. UT: OR = 65.38, 95%CrI = 5.49–45.4);Acupuncture + cupping (AC_CUP vs. UT: OR = 57.48, 95%CrI = 8.11–1764.02); Acupuncture + moxibustion + cupping (AC_MOX_CUP vs. UT: OR = 4.07, 95%CrI = 1.27–15.98); Acupuncture + warm needle moxibustion (AC_WN vs. UT: OR = 19.34, 95%CrI = 4.84–102.68); Floating needle (FN vs. UT: OR = 9.19, 95%CrI = 2.25–63.93); Moxibustion (MOX vs. UT: OR = 3.00, 95%CrI = 2.03–4.51); Moxibustion + acupoint application therapy (MOX_AAT vs. UT: OR = 5.68, 95%CrI = 1.19–45.33); Moxibustion + acupuncture (MOX_AC vs. UT: OR = 19.47, 95%CrI = 9.53–43.04); Moxibustion + warm needle moxibustion (MOX_WN vs. UT: OR = 68.03, 95%CrI = 10.08–2156.00); Traditional Chinese medicine + warm needle moxibustion (TCM_WN vs. UT: OR = 16.15, 95%CrI = 5.95–48.54);Acupuncture (AC vs. UT: OR = 5.00, 95%CrI = 3.81–6.61); Warm needle moxibustion (WN vs. UT: OR = 0.23, 95%CrI = 0.14–0.34)

Compared to Moxibustion + warm needle moxibustion (MOX_WN), the following interventions showed significantly lower effectiveness: Acupoint application therapy (AAT vs. MOX_WN: OR = 0.07, 95%CrI = 0–0.49);Acupuncture (AC vs. MOX_WN: OR = 0.07, 95%CrI = 0–0.51);Acupuncture + moxibustion + cupping (AC_MOX_CUP vs. MOX_WN: OR = 0.06, 95%CrI = 0–0.63); Warm needle moxibustion and moxibustion (WN vs. MOX_WN: OR = 15.18, 95%CrI = 2.4–472.14) (see Table 3 for details).

#### 3.6.2 F2 (IL-2)

Compared to CT (UT), acupoint injection (AI) significantly increased IL-2 levels (AI vs. UT: MD = −8.6, 95%CrI = −18.96 to –0.06).

Compared to acupuncture (AC), acupoint injection (AI) also significantly increased IL-2 (AI vs. AC: MD = −8.6, 95%CrI = −18.96 to −0.06) (see Table 4).

#### 3.6.3 F3 (TNF-α)

Compared with acupoint injection (AI), patients with chronic pelvic inflammatory disease were more likely to receive cupping after acupuncture [AC vs. AI: MD = 27.31, 95%CrI = 7.85, 46.82)], Acupoint application therapy[AAT vs. AI: MD = 22.05, 95%CrI = 1.01, 43.11)], acupuncture + acupoint injection [AC_AI vs. AI: MD = 31.74, and 95%CrI = 0.95, 62.57)], acupuncture + cupping [AC_CUP vs. AI: MD = 32.05, 95%CrI = 1.29, 63.07)], moxibustion [MOX vs. AI: MD = −25.86, 95%CrI = −46.86, −4.8)], and moxibustion + acupoint application therapy [MOX_AAT vs. AI: MD = −31.98, 95%CrI = −62.82, −1.2)], moxibustion + acupuncture [MOX_AC vs. AI: MD = −31.56, 95%CrI = −62.41, −0.6)], and warm needle moxibustion [WN vs. AI: MD = −26.86, 95%CrI = −47.89, −5.8)] treatment resulted in a significant increase in TNF-α.

IL-2 decreased significantly in patients with chronic pelvic inflammatory disease after treatment with acupoint injections [AI vs. UT: MD = −32.4, 95%CrI = −50.13, −14.59)] compared to conventional treatment (UT) (see Supplementary Table 2).

#### 3.6.4 F12 (IL-6)

Compared to Moxibustion + Acupuncture (MOX_AC), acupoint injection (AI) significantly increased IL−6 (AI vs. MOX_AC: MD = 253.59, 95%CrI = 61.51–448.84).

Compared to CT (UT), Moxibustion + Acupuncture (MOX_AC) significantly decreased IL-6 (MOX_AC vs. UT: MD = −180.7, 95%CrI = −340.82 to −21.17) (see Supplementary Table 3).

#### 3.6.5 F13 (CRP)

Compared to Acupoint Injection (AI), the following interventions significantly decreased CRP: Acupoint application therapy (AAT vs. AI: MD = −17.22, 95%CrI = −25.41 to −9.01); Acupoint application therapy + ultrasonic drug penetration (AAT_UDD vs. AI: MD = 16.34, 95%CrI = 5.18–27.54);Acupuncture (AC vs. AI: MD = 16.98, 95%CrI = 8.85–25.13);Acupuncture + cupping (AC_CUP vs. AI: MD = 17.37, 95%CrI = 6.61–28.15);Floating needle (FN vs. AI: MD = −19.78, 95%CrI = −29.09 to −10.46);Moxibustion + acupoint application therapy (MOX_AAT vs. AI: MD = −18.93, 95%CrI = −29.69 to −8.12); Moxibustion + needling (MOX_AC vs. AI: MD = −18.48, 95%CrI = −27.81– −9.16); Warm needle moxibustion (WN vs. AI: MD = −18.38, 95%CrI = −26.90 to −9.88); Warm needle moxibustion + traditional Chinese medicine (WN_TCM vs. AI: MD = −15.00, 95%CrI = −26.90 to −9.88)

Compared to CT (UT), the following significantly decreased CRP: Acupoint application therapy (AAT vs. UT: MD = −3.39, 95%CrI = −6.47 to −0.27); Acupoint injection (AI vs. UT: MD = −20.60, 95%CrI = −28.22 to –12.96); Moxibustion (MOX vs. UT: MD = −13.44, 95%CrI = −17.90 to −8.99) (see Supplementary Table 4).

### 3.7 Cumulative ranking probability results

The interventions were ranked based on the SUCRA results (see Supplementary Table 5).

F1 (Overall Response Rate): MOX_WN (89.1%) > AAT_UDD (88.0%) > AC_CUP (86.8%) > MOX_AC (72.2%) > AC_WN (71.0%) > FN (53.3%) > MOX (15.0%) > AC (36.3%) > WN (30.4%) > UT (0.2%).

F2 (IL-2): UT (66.2%) > AC (61.6%) > MOX (58.6%) > WN (55.4%) > AC_AI (57.1%) > AAT (46.4%) > AC_CUP (4.7%).

F3 (TNF-α): AI (95.8%) > AAT_UDD (82.0%) > MOX (51.6%) > AC_CUP (34.0%) > AC (46.7%) > MOX_AC (35.4%) > WN (47.95%) > UT (27.4%).

F12 (IL-6): MOX_AC (92.6%) > MOX (79.0%) > AAT_UDD (41.0%) > AC_WN (57.3%) > AC_CUP (58.5%) > AC (46.3%) > WN (56.1%) > UT (32.0%)

F13 (CRP): AI (99.4%) > MOX (90.5%) > AAT_UDD (53.0%) > MOX_AC (36.9%) > WN_TCM (61.5%) > AC (52.5%) > FN (25.6%) > WN (37.3%) > UT (13.5%).

**FIGURE 5:**
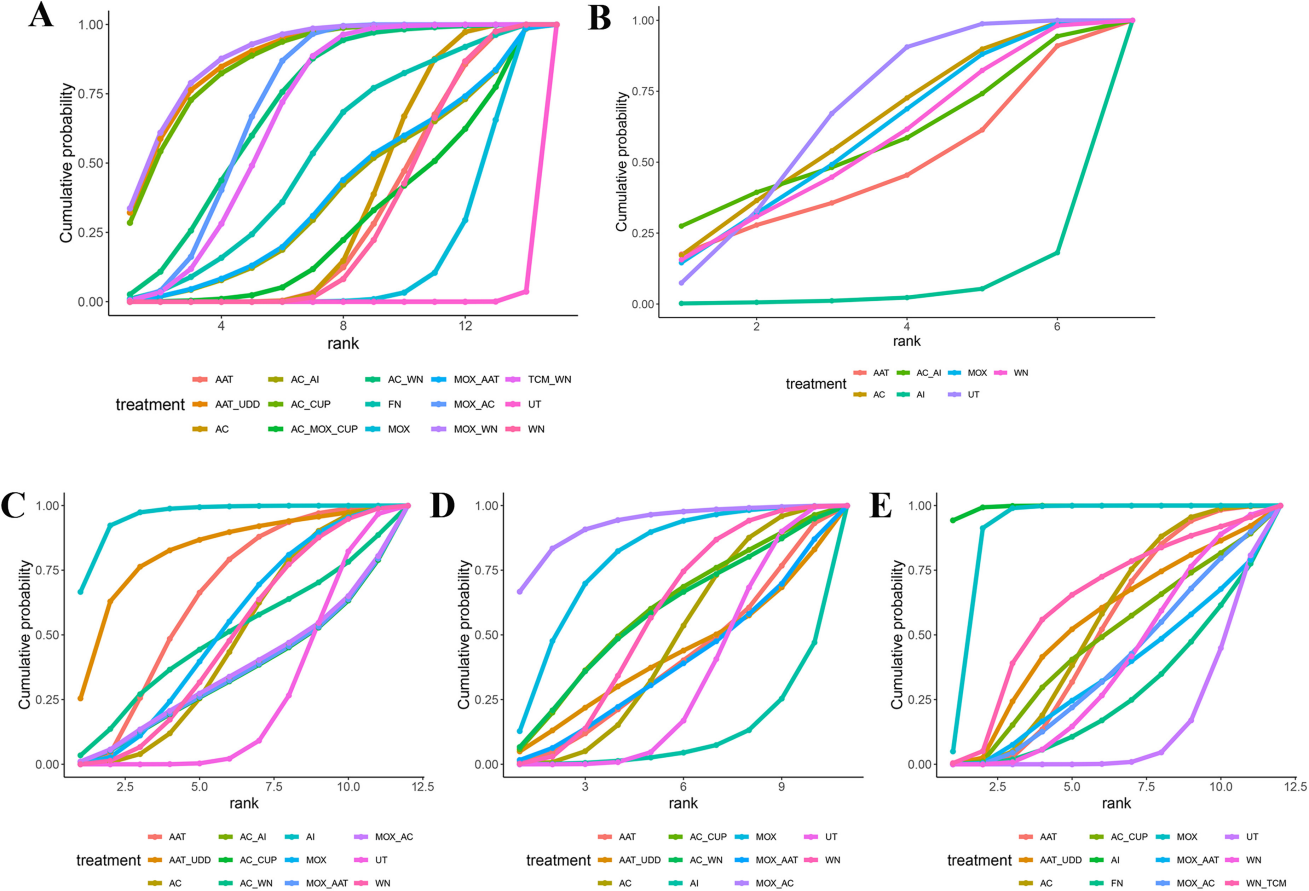
Cumulative probability line graph. **(A)** Efficacy. **(B**) IL-2. **(C)** TNF-α. **(D)** IL-6. **(E)** CRP.

In the cumulative probability line chart, the higher the SUCRA value for a particular therapy, the higher its efficacy ranking. The SUCRA rankings for the efficacy of different acupuncture therapies in improving various outcome measures for chronic pelvic inflammation are as follows (see Figure 5):

F1 ranking: TCM_WN (0.75) > AC_WN (0.70) > MOX_AAT (0.65) > AC_AI (0.60) > AAT (0.55) > MOX_AC (0.50) > UT (0.45) > AC_CUP (0.40) > AAT_UDD (0.35) > FN (0.30) > G (0.25) > AC_MOX_CUP (0.20) > MOX (0.18) > WN (0.15) > MOX_WN (0.12) > AC (0.10).

F2 ranking: MOX (0.75) > AAT (0.65) > AC_AI (0.58) > WN (0.50) > AI (0.35) > AC (0.25) > UT (0.10).

F3 ranking: AC_AI (0.82) > AAT (0.75) > AI (0.68) > MOX_AC (0.55) > AAT_UDD (0.48) > AC_CUP (0.42) > MOX (0.35) > UT (0.28) > G (0.22) > AC_WN (0.18) > MOX_AAT (0.15) > WN (0.12) > AC (0.10).

F12 sorted as: AC_CUP (0.78) > AAT (0.70) > MOX (0.65) > UT (0.58) > WN (0.50) > G (0.42) > AAT_UDD (0.35) > AC_WN (0.28) > MOX_AAT (0.22) > MOX_AC (0.18) > AC (0.15) > AI (0.10).

F13 ranking: AC_CUP (0.85) > UT (0.78) > MOX (0.72) > AAT (0.65) > AI (0.58) > AAT_UDD (0.50) > G (0.42) > MOX_AAT (0.35) > WN (0.28) > WN_TCM (0.22) > MOX_AC (0.18) > FN (0.15) > AC (0.10).

### 3.8 Publication bias analysis

F1 (Effectiveness): Points are scattered without obvious asymmetry, suggesting low publication bias risk.

F2 (IL-2): Fewer points on the right side (positive effect size area) may indicate missing negative results (potential publication bias).

F3 (TNF-α): Symmetrical point distribution, suggesting low bias risk.

F12 (IL-6): Dense clustering on the left side (negative effect size area) may suggest concentration of negative results in small sample studies.

F13 (CRP): Points are generally symmetrical at the top, with few outliers at the bottom, suggesting low overall bias risk. See Supplementary Figure 1 for funnel plots.

## 4 Discussion

By integrating data from 84 randomized controlled trials (RCTs) involving 8147 patients with chronic pelvic inflammatory disease (CPID), this network meta-analysis systematically compared the efficacy of nine interventions, including moxibustion (MOX), acupuncture (AC), traditional Chinese medicine (TCM), acupoint application therapy (AAT), ultrasonic drug penetration (UDD), floating needle (FN), warm needle moxibustion (WN), acupoint injection (AI), and cupping (CUP).(The aforementioned interventions are combined in pairs or multiples to form composite treatment protocols based on clinical needs). Key findings revealed that: AI demonstrated optimal efficacy for modulating TNF-α (SUCRA = 95.8%) and CRP (SUCRA = 99.4%). MOX + AC showed superior improvement in IL-6 (SUCRA = 92.6%). AC achieved the most significant reduction in IL-2 (SUCRA = 61.6%). Moxibustion combined with warm needle moxibustion (MOX + WN) and AAT + UDD ranked highest for overall clinical effectiveness (SUCRA = 89.1 and 88.0%, respectively).

As the first study to establish a multi-dimensional efficacy evaluation system using Bayesian modeling, we quantified the specific advantages of interventions across clinical and inflammatory endpoints—overcoming the single-endpoint limitations of traditional meta-analyses. This approach integrated direct and indirect evidence to establish an “intervention–inflammatory target” precision-matching model, providing an evidence-based foundation for individualized CPID therapy.

### 4.1 Efficacy and mechanism from a traditional Chinese medicine perspective

According to Traditional Chinese Medicine (TCM), chronic pelvic inflammatory disease (CPID) is fundamentally characterized by the accumulation of damp-heat and blood stasis in the lower jiao, which disrupts the normal circulation of qi and blood. This pathological pattern aligns with the classical TCM principle that “obstruction leads to pain,” and helps explain the persistent lower abdominal discomfort, distending pain, and lumbosacral soreness commonly observed in CPID patients.

Among the interventions examined in this study, moxibustion combined with warm needle therapy demonstrated particularly strong therapeutic effects—a finding consistent with the TCM treatment strategy of “warming the meridians to unblock obstruction and promoting blood flow to relieve pain.” Moxibustion acts to warm and strengthen the lower jiao, effectively dispelling cold-dampness, while warm needle therapy directs thermal stimulation deeply into affected tissues. Together, these methods enhance qi and blood circulation, directly addressing the stasis and cold-dampness that underlie CPID pathology.

In terms of acupoint selection, the treatment protocols commonly adopted in the included studies emphasized the use of core points such as Guanyuan (CV4), Qihai (CV6), and Sanyinjiao (SP6). Guanyuan and Qihai, located on the Conception Vessel (Ren Mai), function to warm and tonify the lower jiao. Sanyinjiao, a key point of the Spleen Meridian, helps regulate the functions of the Liver, Spleen, and Kidney. When combined with local points such as Zhongji (CV3) and the extra point Uterus (CA-CV), this combination reflects a treatment strategy that integrates systemic regulation with targeted local intervention.

It is also noteworthy that the analgesic effect of acupuncture is not limited to CPID. Systematic reviews have confirmed its efficacy in managing various chronic pain conditions (99), supporting the broader relevance of our findings. This suggests that acupuncture’s ability to regulate qi and blood circulation represents a shared mechanism that contributes to its therapeutic effects in CPID and other chronic inflammatory disorders.

This study not only confirms the clinical value of specific acupuncture and moxibustion therapies for CPID but also interprets their mechanisms of action through the lens of TCM theory. By integrating classical TCM knowledge with modern clinical evidence, this work helps to establish a more coherent and evidence-informed understanding of how acupuncture treats CPID.

### 4.2 Interpretation of results

#### 4.2.1 Clinical efficacy

MOX + WN (SUCRA = 89.1%) and AAT + UDD (SUCRA = 88.0%) significantly outperformed monotherapies (e.g., AC alone: SUCRA = 36.3%). This synergy arises from: Moxibustion attenuating inflammation via the NLRP3/Caspase-1/GSDMD pathway (100). Warm needle therapy enhancing microcirculation and inflammatory mediator clearance through PI3K/AKT/mTOR signaling (101). Ultrasound drug delivery improving immune function via localized blood flow and anti-inflammatory absorption (102). Acupoint application induces the regulation of IL-6 and IL-10 by ovalbumin (OVA) to alleviate inflammation and achieve local anti-inflammatory effects (103).

#### 4.2.2 Inflammatory response

In this study, IL-2, TNF-α, IL-6, and CRP were selected as key inflammatory markers because they are closely related to the pathological mechanisms of chronic pelvic inflammatory disease (CPID): the core of CPID is persistent inflammatory response, and these four markers can comprehensively assess the inflammatory status by reflecting the degree of disease activity from the dimensions of immune regulation, pro-inflammatory response, and acute inflammation, respectively. They have been widely demonstrated to correlate with disease severity, treatment efficacy and prognosis in pelvic inflammatory diseases, and are commonly used in international and national studies to evaluate inflammation, facilitating cross-sectional comparison and interpretation of results.

IL-2 is mainly secreted by T cells, which can promote the proliferation of immune cells and enhance the immune response, and changes in its level can reflect the dynamic changes of the body’s immune regulation function in inflammation. TNF-α is an important pro-inflammatory cytokine, which can initiate the inflammatory cascade reaction and induce the release of other inflammatory factors, and plays a key role in the development of pelvic inflammatory injuries. IL-6 is a combination of proinflammatory and anti-inflammatory effects, and persists in chronic inflammation, and has been shown to play a role in both inflammation and anti-inflammatory diseases. IL-6 has both pro-inflammatory and anti-inflammatory effects, and is continuously elevated in chronic inflammation, correlating with tissue damage, pain and inflammation prolongation, and is a sensitive indicator of the degree of active inflammation. CRP is an acute phase protein synthesized by the liver, and is rapidly elevated in response to inflammatory stimuli, which can reflect the intensity of the body’s acute inflammatory response, and is often used to assess the effect of inflammation control after treatment. Therefore, changes in IL-2, IL-6, TNF-α and CRP levels were evaluated as indicators of inflammation improvement.

IL-2 Reduction: AC (SUCRA = 61.6%) outperformed conventional therapy (UT; SUCRA = 66.2%), likely due to UT’s insufficient suppression of baseline inflammation. AC’s immunomodulatory role involves inhibiting TLR4/NF-κB signaling (104–106). TNF-α/CRP Suppression: AI ranked first for both (SUCRA > 95%), attributable to localized drug effects at acupoints (107, 108). IL-6 Modulation: MOX + AC (SUCRA = 92.6%; MD = 253.59, 95% CI: 61.51–448.84) leverages synergistic warming (via PI3K/AKT/mTOR) and needling effects (109–111). Electroacupuncture further suppresses inflammation through neural pathways (112, 113).

### 4.3 Comparison with prior reviews

Unlike previous reviews focused solely on antibiotics, this study integrated nine acupuncture therapies and four inflammatory biomarkers into a coherent evidence network, establishing a “therapy–target” association framework. Using Bayesian network meta-analysis with SUCRA ranking, we clarified the efficacy hierarchy of acupuncture therapies in inflammation control, overcoming the limitations of qualitative comparisons. Furthermore, we identified the “MOX + AC → IL-6 → PI3K/AKT/mTOR” pathway, extending evidence on acupuncture’s anti-inflammatory mechanisms. The model showed excellent convergence (PSRF = 1.00), consistent with the approach of Fan Y, Zhu C, etc.(114), confirming methodological robustness.

### 4.4 Clinical implications

Unlike previous reviews that primarily focused on antibiotic treatments, the present study integrated nine acupuncture-related therapies and four inflammatory biomarkers into a comprehensive evidence network, thereby establishing a more holistic evidence base for “therapy–target” associations. By adopting a Bayesian framework for network meta-analysis, this study provided quantitative rankings of interventions based on the surface under the cumulative ranking curve (SUCRA), overcoming the qualitative limitations of conventional pairwise meta-analyses and clarifying the efficacy hierarchy of different acupuncture modalities in modulating inflammatory responses. Furthermore, the identification of the “moxibustion plus acupuncture → IL-6 → PI3K/AKT/mTOR signaling pathway” axis adds a new dimension to the mechanistic understanding of acupuncture’s anti-inflammatory effects. The model demonstrated excellent convergence (PSRF = 1.00), which aligns with the analytical approach employed by Fan et al. (2) in a study on acute gouty arthritis, underscoring the methodological robustness and reliability of our findings.

### 4.5 Limitations

Despite a robust evidence network and model convergence (PSRF = 1.00), limitations include: methodological Constraints: blinding challenges in acupuncture trials and inadequate reporting of allocation concealment may introduce bias. Evidence Gaps: limited RCTs for interventions like floating needles or cupping weaken network confidence. Statistical Inconsistency: direct/indirect comparison discrepancies for IL-6 and CRP require head-to-head RCT validation. Heterogeneity: variability in acupuncture duration/point selection (e.g., IL-2, TNF-α) warrants subgroup exploration. Generalizability: short intervention periods (2–4 weeks) and non-standardized techniques limit extrapolation.

## 5 Conclusion

This Bayesian network meta-analysis establishes optimal CPID therapies: overall Efficacy: MOX + WN or AAT + UDD (SUCRA > 88%). Targeted Modulation: TNF-α/CRP → AI (SUCRA > 95%). IL-6 → MOX + AC (SUCRA = 92.6%). IL-2 → AC (SUCRA = 61.6%). Clinical decisions should align inflammatory profiles with “therapy–target”*matching (e.g., AI for antibiotic resistance). Future multicenter RCTs should validate combination therapies and molecular targets to standardize acupuncture in chronic inflammatory disease management.

## Data Availability

The original contributions presented in the study are included in the article/Supplementary material, further inquiries can be directed to the corresponding authors.
