# Supplementary material for: The efficacy of acupuncture and related treatments on chronic pelvic inflammatory disease: a network meta-analysis
Source: Front Med (Lausanne). 2026 Jan 12;12:1731543. doi: 10.3389/fmed.2025.1731543 (PMC12805501; doi:10.3389/fmed.2025.1731543)
Supplement: Supplementary file 1 [file Data_Sheet_1.docx]

**Supplementary Materials**

**Supplementary Table 1** The search strategy

**(Pubmed)**

| Search number | Query | Results |
| --- | --- | --- |
| 1 | Pelvic Inflammatory Disease[MeSH Terms] | 11858 |
| 2 | "pelvic inflammatory disease"[Title/Abstract] OR "disease pelvic inflammatory"[Title/Abstract] OR "diseases pelvic inflammatory"[Title/Abstract] OR "inflammatory diseases pelvic"[Title/Abstract] OR "pelvic inflammatory diseases"[Title/Abstract] OR "inflammatory disease pelvic"[Title/Abstract] OR "inflammatory pelvic disease"[Title/Abstract] OR (("Disease"[MeSH Terms] OR "Disease"[All Fields] OR "Diseases"[All Fields] OR "disease s"[All Fields] OR "diseased"[All Fields]) AND "inflammatory pelvic"[Title/Abstract]) OR (("Disease"[MeSH Terms] OR "Disease"[All Fields] OR "Diseases"[All Fields] OR "disease s"[All Fields] OR "diseased"[All Fields]) AND "inflammatory pelvic"[Title/Abstract]) OR "inflammatory pelvic diseases"[Title/Abstract] OR (("pelvics"[All Fields] OR "pelvis"[MeSH Terms] OR "pelvis"[All Fields] OR "Pelvic"[All Fields]) AND "diseases inflammatory"[Title/Abstract]) OR (("pelvics"[All Fields] OR "pelvis"[MeSH Terms] OR "pelvis"[All Fields] OR "Pelvic"[All Fields]) AND "disease inflammatory"[Title/Abstract]) OR "Adnexitis"[Title/Abstract] | 6069 |
| 3 | "pelvic inflammatory disease"[MeSH Terms] OR ("pelvic inflammatory disease"[Title/Abstract] OR "disease pelvic inflammatory"[Title/Abstract] OR "diseases pelvic inflammatory"[Title/Abstract] OR "inflammatory diseases pelvic"[Title/Abstract] OR "pelvic inflammatory diseases"[Title/Abstract] OR "inflammatory disease pelvic"[Title/Abstract] OR "inflammatory pelvic disease"[Title/Abstract] OR (("Disease"[MeSH Terms] OR "Disease"[All Fields] OR "Diseases"[All Fields] OR "disease s"[All Fields] OR "diseased"[All Fields]) AND "inflammatory pelvic"[Title/Abstract]) OR (("Disease"[MeSH Terms] OR "Disease"[All Fields] OR "Diseases"[All Fields] OR "disease s"[All Fields] OR "diseased"[All Fields]) AND "inflammatory pelvic"[Title/Abstract]) OR "inflammatory pelvic diseases"[Title/Abstract] OR (("pelvics"[All Fields] OR "pelvis"[MeSH Terms] OR "pelvis"[All Fields] OR "Pelvic"[All Fields]) AND "diseases inflammatory"[Title/Abstract]) OR (("pelvics"[All Fields] OR "pelvis"[MeSH Terms] OR "pelvis"[All Fields] OR "Pelvic"[All Fields]) AND "disease inflammatory"[Title/Abstract]) OR "Adnexitis"[Title/Abstract]) | 14749 |
| 4 | "acupuncture"[MeSH Terms] OR "acupuncture therapy"[MeSH Terms] OR "acupuncture therapy"[MeSH Terms] OR "acupuncture, ear"[MeSH Terms] OR "acupuncture points"[MeSH Terms] OR "electroacupuncture"[MeSH Terms] OR "moxibustion"[MeSH Terms] | 32179 |
| 5 | "Acupuncture"[Title/Abstract] OR "Pharmacopuncture"[Title/Abstract] OR "acupuncture therapy"[Title/Abstract] OR "acupuncture treatment"[Title/Abstract] OR "acupuncture treatments"[Title/Abstract] OR "treatment acupuncture"[Title/Abstract] OR "therapy acupuncture"[Title/Abstract] OR "pharmacoacupuncture treatment"[Title/Abstract] OR (("therapeutics"[MeSH Terms] OR "therapeutics"[All Fields] OR "Treatments"[All Fields] OR "Therapy"[MeSH Subheading] OR "Therapy"[All Fields] OR "Treatment"[All Fields] OR "treatment s"[All Fields]) AND "Pharmacoacupuncture"[Title/Abstract]) OR "pharmacoacupuncture therapy"[Title/Abstract] OR (("therapeutics"[MeSH Terms] OR "therapeutics"[All Fields] OR "therapies"[All Fields] OR "Therapy"[MeSH Subheading] OR "Therapy"[All Fields] OR "therapy s"[All Fields] OR "therapys"[All Fields]) AND "Pharmacoacupuncture"[Title/Abstract]) OR "Acupotomy"[Title/Abstract] OR "Acupotomies"[Title/Abstract] OR "acupuncture ear"[Title/Abstract] OR (("acupunctural"[All Fields] OR "Acupuncture"[MeSH Terms] OR "Acupuncture"[All Fields] OR "acupuncture therapy"[MeSH Terms] OR ("Acupuncture"[All Fields] AND "Therapy"[All Fields]) OR "acupuncture therapy"[All Fields] OR "acupuncture s"[All Fields] OR "acupunctured"[All Fields] OR "Acupunctures"[All Fields] OR "acupuncturing"[All Fields]) AND "Ear"[Title/Abstract]) OR (("Ear"[MeSH Terms] OR "Ear"[All Fields]) AND "Acupunctures"[Title/Abstract]) OR "acupuncture auricular"[Title/Abstract] OR (("acupunctural"[All Fields] OR "Acupuncture"[MeSH Terms] OR "Acupuncture"[All Fields] OR "acupuncture therapy"[MeSH Terms] OR ("Acupuncture"[All Fields] AND "Therapy"[All Fields]) OR "acupuncture therapy"[All Fields] OR "acupuncture s"[All Fields] OR "acupunctured"[All Fields] OR "Acupunctures"[All Fields] OR "acupuncturing"[All Fields]) AND "Auricular"[Title/Abstract]) OR "auricular acupunctures"[Title/Abstract] OR "auricular acupuncture"[Title/Abstract] OR "ear acupuncture"[Title/Abstract] OR "acupuncture points"[Title/Abstract] OR "acupuncture point"[Title/Abstract] OR "point acupuncture"[Title/Abstract] OR "points acupuncture"[Title/Abstract] OR "Acupoints"[Title/Abstract] OR "Acupoint"[Title/Abstract] OR "Electroacupuncture"[Title/Abstract] OR "Moxibustion"[Title/Abstract] OR "Moxabustion"[Title/Abstract] | 38243 |
| 6 | "Acupuncture"[MeSH Terms] OR "acupuncture therapy"[MeSH Terms] OR "acupuncture therapy"[MeSH Terms] OR "acupuncture, ear"[MeSH Terms] OR "acupuncture points"[MeSH Terms] OR "Electroacupuncture"[MeSH Terms] OR "Moxibustion"[MeSH Terms] OR ("Acupuncture"[Title/Abstract] OR "Pharmacopuncture"[Title/Abstract] OR "acupuncture therapy"[Title/Abstract] OR "acupuncture treatment"[Title/Abstract] OR "acupuncture treatments"[Title/Abstract] OR "treatment acupuncture"[Title/Abstract] OR "therapy acupuncture"[Title/Abstract] OR "pharmacoacupuncture treatment"[Title/Abstract] OR (("therapeutics"[MeSH Terms] OR "therapeutics"[All Fields] OR "Treatments"[All Fields] OR "Therapy"[MeSH Subheading] OR "Therapy"[All Fields] OR "Treatment"[All Fields] OR "treatment s"[All Fields]) AND "Pharmacoacupuncture"[Title/Abstract]) OR "pharmacoacupuncture therapy"[Title/Abstract] OR (("therapeutics"[MeSH Terms] OR "therapeutics"[All Fields] OR "therapies"[All Fields] OR "Therapy"[MeSH Subheading] OR "Therapy"[All Fields] OR "therapy s"[All Fields] OR "therapys"[All Fields]) AND "Pharmacoacupuncture"[Title/Abstract]) OR "Acupotomy"[Title/Abstract] OR "Acupotomies"[Title/Abstract] OR "acupuncture ear"[Title/Abstract] OR (("acupunctural"[All Fields] OR "Acupuncture"[MeSH Terms] OR "Acupuncture"[All Fields] OR "acupuncture therapy"[MeSH Terms] OR ("Acupuncture"[All Fields] AND "Therapy"[All Fields]) OR "acupuncture therapy"[All Fields] OR "acupuncture s"[All Fields] OR "acupunctured"[All Fields] OR "Acupunctures"[All Fields] OR "acupuncturing"[All Fields]) AND "Ear"[Title/Abstract]) OR (("Ear"[MeSH Terms] OR "Ear"[All Fields]) AND "Acupunctures"[Title/Abstract]) OR "acupuncture auricular"[Title/Abstract] OR (("acupunctural"[All Fields] OR "Acupuncture"[MeSH Terms] OR "Acupuncture"[All Fields] OR "acupuncture therapy"[MeSH Terms] OR ("Acupuncture"[All Fields] AND "Therapy"[All Fields]) OR "acupuncture therapy"[All Fields] OR "acupuncture s"[All Fields] OR "acupunctured"[All Fields] OR "Acupunctures"[All Fields] OR "acupuncturing"[All Fields]) AND "Auricular"[Title/Abstract]) OR "auricular acupunctures"[Title/Abstract] OR "auricular acupuncture"[Title/Abstract] OR "ear acupuncture"[Title/Abstract] OR "acupuncture points"[Title/Abstract] OR "acupuncture point"[Title/Abstract] OR "point acupuncture"[Title/Abstract] OR "points acupuncture"[Title/Abstract] OR "Acupoints"[Title/Abstract] OR "Acupoint"[Title/Abstract] OR "Electroacupuncture"[Title/Abstract] OR "Moxibustion"[Title/Abstract] OR "Moxabustion"[Title/Abstract]) | 42793 |
| 7 | #3 AND #6 | 54 |

**(Embase)**

| Search number | Query | Results |
| --- | --- | --- |
| 1 | 'pelvic inflammatory disease'/exp | 27724 |
| 2 | 'disease, pelvic inflammatory':ab,ti | 32 |
| 3 | 'pelvic inflammatory disease':ab,ti | 6130 |
| 4 | 'diseases, pelvic inflammatory':ab,ti | 13 |
| 5 | 'diseases, pelvic inflammatory':ab,ti | 2 |
| 6 | 'pelvic inflammatory diseases':ab,ti | 292 |
| 7 | 'inflammatory disease, pelvic':ab,ti | 22 |
| 8 | 'inflammatory pelvic disease':ab,ti | 49 |
| 9 | 'disease, inflammatory pelvic':ab,ti | 0 |
| 10 | 'diseases, inflammatory pelvic':ab,ti | 1 |
| 11 | 'inflammatory pelvic diseases':ab,ti | 9 |
| 12 | 'pelvic diseases, inflammatory':ab,ti | 0 |
| 13 | 'pelvic disease, inflammatory':ab,ti | 0 |
| 14 | 'adnexitis':ab,ti | 563 |
| 15 | #1 OR #2 OR #3 OR #4 OR #5 OR #6 OR #7 OR #8 OR #9 OR #10 OR #11 OR #12 OR #13 OR #14 | 29578 |
| 16 | 'acupuncture'/exp | 62771 |
| 17 | 'auricular acupuncture'/exp | 1062 |
| 18 | 'acupuncture point'/exp | 7061 |
| 19 | 'electroacupuncture'/exp | 10631 |
| 20 | 'moxibustion'/exp | 5287 |
| 21 | 'acupuncture':ab,ti | 41789 |
| 22 | 'pharmacopuncture':ab,ti | 385 |
| 23 | 'acupuncture therapy':ab,ti | 2320 |
| 24 | 'acupuncture treatment':ab,ti | 2513 |
| 25 | 'acupuncture treatments':ab,ti | 857 |
| 26 | 'treatment, acupuncture':ab,ti | 270 |
| 27 | 'therapy, acupuncture':ab,ti | 362 |
| 28 | 'pharmacoacupuncture treatment':ab,ti | 1 |
| 29 | 'treatment, pharmacoacupuncture':ab,ti | 0 |
| 30 | 'pharmacoacupuncture therapy':ab,ti | 3 |
| 31 | 'therapy, pharmacoacupuncture':ab,ti | 0 |
| 32 | 'acupotomy':ab,ti | 220 |
| 33 | 'aacupotomies':ab,ti | 0 |
| 34 | 'acupuncture, ear':ab,ti | 36 |
| 35 | 'acupunctures, ear':ab,ti | 0 |
| 36 | 'ear acupunctures':ab,ti | 0 |
| 37 | 'acupuncture, auricular':ab,ti | 43 |
| 38 | 'acupunctures, auricular':ab,ti | 0 |
| 39 | 'auricular acupunctures':ab,ti | 4 |
| 40 | 'auricular acupuncture':ab,ti | 767 |
| 41 | 'ear acupuncture':ab,ti | 381 |
| 42 | 'acupuncture points':ab,ti | 2417 |
| 43 | 'acupuncture point':ab,ti | 1077 |
| 44 | 'point, acupuncture':ab,ti | 139 |
| 45 | 'points, acupuncture':ab,ti | 49 |
| 46 | 'acupoints':ab,ti | 6052 |
| 47 | 'acupoint':ab,ti | 5503 |
| 48 | 'electroacupuncture':ab,ti | 9240 |
| 49 | 'moxibustion':ab,ti | 5144 |
| 50 | 'moxabustion':ab,ti | 2 |
| 51 | #16 OR #17 OR #18 OR #19 OR #20 OR #21 OR #22 OR #23 OR #24 OR #25 OR #26 OR #27 OR #28 OR #29 OR #30 OR #31 OR #32 OR #33 OR #34 OR #35 OR #36 OR #37 OR #38 OR #39 OR #40 OR #41 OR #42 OR #43 OR #44 OR #45 OR #46 OR #47 OR #48 OR #49 OR #50 | 68916 |
| 52 | #15 AND #51 | 107 |

**(Wed of science)**

| Search number | Query | Results |
| --- | --- | --- |
| 1 | TS=(Pelvic Inflammatory Disease) OR TS=(Disease, Pelvic Inflammatory) OR TS=(Diseases, Pelvic Inflammatory) OR TS=(Inflammatory Diseases, Pelvic) OR TS=(Pelvic Inflammatory Diseases) OR TS=(Inflammatory Disease, Pelvic) OR TS=(Inflammatory Pelvic Disease) OR TS=(Disease, Inflammatory Pelvic) OR TS=(Diseases, Inflammatory Pelvic) OR TS=(Inflammatory Pelvic Diseases) OR TS=(Pelvic Diseases, Inflammatory) OR TS=(Pelvic Disease, Inflammatory) OR TS=(Adnexitis) | 7826 |
| 2 | TS=(Acupuncture) OR TS=(Pharmacopuncture) OR TS=(Acupuncture Therapy) OR TS=(Acupuncture Treatment) OR TS=(Acupuncture Treatments) OR TS=(Treatment, Acupuncture) OR TS=(Therapy, Acupuncture) OR TS=(Pharmacoacupuncture Treatment) OR TS=(Treatment, Pharmacoacupuncture) OR TS=(Pharmacoacupuncture Therapy) OR TS=(Therapy, Pharmacoacupuncture) OR TS=(Acupotomy) OR TS=(Acupotomies) OR TS=(Acupuncture, Ear) OR TS=(Acupunctures, Ear) OR TS=(Ear Acupunctures) OR TS=(Acupuncture, Auricular) OR TS=(Acupunctures, Auricular) OR TS=(Auricular Acupunctures) OR TS=(Auricular Acupuncture) OR TS=(Ear Acupuncture) OR TS=(Acupuncture Points) OR TS=(Acupuncture Point) OR TS=(Point, Acupuncture) OR TS=(Points, Acupuncture) OR TS=(Acupoints) OR TS=(Acupoint) OR TS=(Electroacupuncture) OR TS=(Moxibustion) OR TS=(Moxabustion) | 34037 |
| 3 | #2 AND #1 | 38 |

**(Cochrane Library)**

| Search number | Query | Results |
| --- | --- | --- |
| 1 | MeSH descriptor: [Pelvic Inflammatory Disease] explode all trees | 627 |
| 2 | (Pelvic Inflammatory Disease):ti,ab,kw OR (Disease, Pelvic Inflammatory):ti,ab,kw OR (Diseases, Pelvic Inflammatory):ti,ab,kw OR (Inflammatory Diseases, Pelvic):ti,ab,kw OR (Pelvic Inflammatory Diseases):ti,ab,kw | 1175 |
| 3 | (Inflammatory Disease, Pelvic):ti,ab,kw OR (Inflammatory Pelvic Disease):ti,ab,kw OR (Disease, Inflammatory Pelvic):ti,ab,kw OR (Diseases, Inflammatory Pelvic):ti,ab,kw OR (Inflammatory Pelvic Diseases):ti,ab,kw | 1175 |
| 4 | (Pelvic Diseases, Inflammatory):ti,ab,kw OR (Pelvic Disease, Inflammatory):ti,ab,kw OR (Adnexitis):ti,ab,kw | 1192 |
| 5 | #1or#2or#3or#4 | 1533 |
| 6 | MeSH descriptor: [Acupuncture] explode all trees | 217 |
| 7 | MeSH descriptor: [Acupuncture Therapy] explode all trees | 7083 |
| 8 | MeSH descriptor: [Acupuncture, Ear] explode all trees | 266 |
| 9 | MeSH descriptor: [Acupuncture Points] explode all trees | 2790 |
| 10 | MeSH descriptor: [Electroacupuncture] explode all trees | 1173 |
| 11 | MeSH descriptor: [Moxibustion] explode all trees | 684 |
| 12 | (Acupuncture):ti,ab,kw OR (Pharmacopuncture):ti,ab,kw OR (Acupuncture Therapy):ti,ab,kw OR (Acupuncture Treatment):ti,ab,kw OR (Acupuncture Treatments):ti,ab,kw | 21066 |
| 13 | (Treatment, Acupuncture):ti,ab,kw OR (Therapy, Acupuncture):ti,ab,kw OR (Pharmacoacupuncture Treatment):ti,ab,kw OR (Treatment, Pharmacoacupuncture):ti,ab,kw OR (Pharmacoacupuncture Therapy):ti,ab,kw | 16872 |
| 14 | (Therapy, Pharmacoacupuncture):ti,ab,kw OR (Acupotomy):ti,ab,kw OR (Acupotomies):ti,ab,kw OR (Acupuncture, Ear):ti,ab,kw OR (Acupunctures, Ear):ti,ab,kw | 1086 |
| 15 | (Ear Acupunctures):ti,ab,kw OR (Acupuncture, Auricular):ti,ab,kw OR (Acupunctures, Auricular):ti,ab,kw OR (Auricular Acupunctures):ti,ab,kw OR (Auricular Acupuncture):ti,ab,kw | 1050 |
| 16 | (Ear Acupuncture):ti,ab,kw OR (Acupuncture Points):ti,ab,kw OR (Acupuncture Point):ti,ab,kw OR (Point, Acupuncture):ti,ab,kw OR (Points, Acupuncture):ti,ab,kw | 8515 |
| 17 | (Acupoints):ti,ab,kw OR (Acupoint):ti,ab,kw OR (Electroacupuncture):ti,ab,kw OR (Moxibustion):ti,ab,kw OR (Moxabustion):ti,ab,kw | 11261 |
| 18 | (Acupoints):ti,ab,kw OR (Acupoint):ti,ab,kw OR (Electroacupuncture):ti,ab,kw OR (Moxibustion):ti,ab,kw OR (Moxabustion):ti,ab,kw | 25468 |
| 19 | #5and#18 | 38 |

**Supplementary Table 2** Results of network meta-analysis of TNF-α.

| MD95%Cri | | | | | | | | | | | |
| --- | --- | --- | --- | --- | --- | --- | --- | --- | --- | --- | --- |
| AAT |  |  |  |  |  |  |  |  |  |  |  |
| 12.42 (-13.08, 37.94) | AAT_UDD |  |  |  |  |  |  |  |  |  |  |
| -5.27 (-19.06, 8.57) | -17.66 (-46.72, 11.4) | AC |  |  |  |  |  |  |  |  |  |
| -9.7 (-37.11, 18.1) | -22.11 (-59.46, 15.7) | -4.46 (-30.73, 22.08) | AC_AI |  |  |  |  |  |  |  |  |
| -10 (-37.67, 17.53) | -22.37 (-60, 15.12) | -4.78 (-31.15, 21.73) | -0.36 (-36.06, 35.12) | AC_CUP |  |  |  |  |  |  |  |
| -4.1 (-33.97, 25.82) | -16.5 (-55.7, 22.87) | 1.15 (-27.43, 29.87) | 5.63 (-31.8, 42.72) | 5.91 (-31.54, 43.28) | AC_WN |  |  |  |  |  |  |
| 22.05 (1.01, 43.11)* | 9.67 (-23.44, 42.68) | 27.31 (7.85, 46.82)* | 31.74 (0.95, 62.57)* | 32.05 (1.29, 63.07)* | 26.17 (-6.76, 58.8) | AI |  |  |  |  |  |
| -3.78 (-19.68, 12.21) | -16.19 (-46.26, 13.88) | 1.49 (-12.32, 15.3) | 5.93 (-21.65, 33.41) | 6.26 (-21.4, 33.87) | 0.34 (-29.41, 30.14) | -25.86 (-46.86, -4.81) | MOX |  |  |  |  |
| -9.96 (-37.58, 17.68) | -22.25 (-59.92, 15.28) | -4.64 (-31.15, 21.76) | -0.25 (-35.71, 35.39) | 0.16 (-35.55, 35.85) | -5.8 (-43.2, 31.52) | -31.98 (-62.82, -1.2) | -6.13 (-33.85, 21.6) | MOX_AAT |  |  |  |
| -9.44 (-37.1, 18.09) | -21.83 (-59.54, 15.66) | -4.23 (-30.73, 22.28) | 0.24 (-35.39, 35.81) | 0.54 (-35.16, 36.27) | -5.34 (-42.93, 31.95) | -31.56 (-62.41, -0.6) | -5.69 (-33.48, 21.96) | 0.44 (-35.29, 36.18) | MOX_AC |  |  |
| -10.33 (-21.57, 0.98) | -22.72 (-50.62, 5.12) | -5.07 (-12.98, 2.92) | -0.65 (-25.87, 24.51) | -0.3 (-25.51, 24.91) | -6.23 (-33.9, 21.33) | -32.4 (-50.13, -14.59) | -6.54 (-17.81, 4.77) | -0.41 (-25.57, 24.79) | -0.84 (-26.07, 24.51) | UT |  |
| -4.8 (-20.77, 11.22) | -17.17 (-47.22, 12.86) | 0.48 (-13.41, 14.31) | 4.9 (-22.65, 32.36) | 5.22 (-22.4, 32.91) | -0.67 (-25.9, 24.36) | -26.86 (-47.89, -5.8) | -0.99 (-16.99, 15.01) | 5.12 (-22.55, 32.68) | 4.71 (-23.03, 32.44) | 5.53 (-5.77, 16.85) | WN |

*MEANS P＜0.05

**Supplementary Table 3** Results of network meta-analysis of IL-6.

| MD95%Cri | | | | | | | | | | |
| --- | --- | --- | --- | --- | --- | --- | --- | --- | --- | --- |
| AAT |  |  |  |  |  |  |  |  |  |  |
| 1.3 (-148.33, 151.42) | AAT_UDD |  |  |  |  |  |  |  |  |  |
| 17.07 (-142.27, 175.66) | 15.63 (-204.22, 232.84) | AC |  |  |  |  |  |  |  |  |
| 48.56 (-163.82, 261.76) | 47.38 (-213.16, 306.73) | 31.24 (-126.85, 190.54) | AC_CUP |  |  |  |  |  |  |  |
| 46.44 (-177.73, 271.38) | 45.25 (-224.67, 315.15) | 29.4 (-142.71, 202.33) | -2.22 (-225.41, 222.67) | AC_WN |  |  |  |  |  |  |
| -76.7 (-265.53, 106.99) | -77.84 (-320.7, 158.99) | -93.69 (-216.1, 24.86) | -125.22 (-313.84, 58.89) | -123.08 (-323.6, 73.61) | AI |  |  |  |  |  |
| 99.31 (-84.84, 287.16) | 98.44 (-140.75, 338.15) | 82.37 (-36.56, 204.35) | 51.08 (-133.85, 238.06) | 52.86 (-143.25, 253.22) | 176.38 (24.82, 333.05)* | MOX |  |  |  |  |
| -3.41 (-216.28, 208.88) | -4.89 (-265.34, 255.42) | -20.47 (-178.78, 138.14) | -52.1 (-264.08, 160.38) | -49.92 (-274.35, 172.25) | 73.26 (-111.1, 261.25) | -103.07 (-291.03, 81.97) | MOX_AAT |  |  |  |
| 177.02 (-43.01, 396.15) | 175.48 (-92.17, 440.23) | 159.82 (-7.65, 327.15) | 128.62 (-91.38, 347.93) | 130.42 (-99.06, 358.95) | 253.59 (61.51, 448.84)* | 77.56 (-117.35, 268.59) | 180.4 (-39.94, 399.75) | MOX_AC |  |  |
| -3.96 (-155.26, 147.39) | -5.12 (-219.61, 206.97) | -20.82 (-71.08, 29.16) | -52.15 (-202.87, 97.67) | -50.26 (-215.25, 115.02) | 72.95 (-34.97, 184.21) | -103.2 (-214.05, 4.34) | -0.25 (-151.17, 149.92) | -180.7 (-340.82, -21.17) | UT |  |
| 35.8 (-129.62, 201.97) | 34.56 (-189.83, 257.43) | 19.09 (-65.3, 102.94) | -12.41 (-177.37, 152.7) | -10.47 (-160.84, 139.35) | 112.59 (-14.13, 242.59) | -63.47 (-193.94, 63.26) | 39.57 (-125.12, 203.87) | -140.73 (-313.33, 31.98) | 39.87 (-27.54, 107.49) | WN |

*MEANS P＜0.05

**Supplementary Table 4** Results of network meta-analysis of CRP.

| MD95%Cri | | | | | | | | | | | |
| --- | --- | --- | --- | --- | --- | --- | --- | --- | --- | --- | --- |
| AAT |  |  |  |  |  |  |  |  |  |  |  |
| 0.88 (-6.76, 8.51) | AAT_UDD |  |  |  |  |  |  |  |  |  |  |
| 0.24 (-4.01, 4.47) | -0.64 (-9.35, 8.08) | AC |  |  |  |  |  |  |  |  |  |
| -0.16 (-8.41, 8.07) | -1.03 (-12.34, 10.18) | -0.39 (-8.54, 7.75) | AC_CUP |  |  |  |  |  |  |  |  |
| 17.22 (9.01, 25.41)* | 16.34 (5.18, 27.54)* | 16.98 (8.85, 25.13)* | 17.37 (6.61, 28.15)* | AI |  |  |  |  |  |  |  |
| -2.57 (-8.81, 3.65) | -3.45 (-13.33, 6.4) | -2.8 (-8.95, 3.35) | -2.42 (-11.77, 6.98) | -19.78 (-29.09, -10.46)* | FN |  |  |  |  |  |  |
| 10.05 (4.66, 15.5)* | 9.17 (-0.2, 18.54) | 9.81 (4.54, 15.13)* | 10.22 (1.4, 19.06)* | -7.17 (-15.98, 1.68) | 12.62 (5.61, 19.67)* | MOX |  |  |  |  |  |
| -1.73 (-9.88, 6.47) | -2.6 (-13.81, 8.53) | -1.96 (-10.04, 6.13) | -1.56 (-12.25, 9.16) | -18.93 (-29.69, -8.12)* | 0.85 (-8.47, 10.16) | -11.76 (-20.59, -2.97)* | MOX_AAT |  |  |  |  |
| -1.27 (-7.54, 4.95) | -2.15 (-12.02, 7.69) | -1.5 (-7.65, 4.59) | -1.1 (-10.47, 8.27) | -18.48 (-27.81, -9.16)* | 1.3 (-6.38, 8.95) | -11.32 (-18.36, -4.3)* | 0.44 (-8.86, 9.73) | MOX_AC |  |  |  |
| -3.39 (-6.47, -0.27)* | -4.27 (-12.5, 3.97) | -3.62 (-6.51, -0.73)* | -3.24 (-10.85, 4.42) | -20.6 (-28.22, -12.96)* | -0.82 (-6.22, 4.62) | -13.44 (-17.9, -8.99)* | -1.67 (-9.24, 5.89) | -2.12 (-7.52, 3.33) | UT |  |  |
| -1.16 (-6.11, 3.77) | -2.05 (-11.14, 7.05) | -1.4 (-6.2, 3.39) | -1.01 (-9.53, 7.54) | -18.38 (-26.9, -9.88)* | 1.4 (-5.21, 8.06) | -11.22 (-17.12, -5.39)* | 0.55 (-7.96, 9.01) | 0.11 (-6.5, 6.74) | 2.22 (-1.6, 6.04) | WN |  |
| 2.21 (-6.87, 11.32) | 1.34 (-10.56, 13.27) | 1.97 (-7.08, 11.02) | 2.38 (-9.05, 13.87) | -15 (-26.45, -3.59)* | 4.78 (-5.32, 14.95) | -7.82 (-17.49, 1.79) | 3.93 (-7.46, 15.34) | 3.49 (-6.61, 13.61) | 5.59 (-2.94, 14.15) | 3.37 (-4.31, 11.02) | WN_TCM |

*MEANS P＜0.05

**Supplementary Table 5** Cumulative probability.

| Treatment | F1(%) | F2（%） | F3(%) | F12(%) | F13(%) |
| --- | --- | --- | --- | --- | --- |
| AAT | 31.5% | 46.4% | 63.8% | 39.0% | 49.9% |
| AAT_UDD | 88.0% | NR | 82.0% | 41.0% | 53.0% |
| AC | 36.3% | 61.6% | 46.7% | 46.3% | 52.5% |
| AC_AI | 39.1% | 57.1% | 34.9% | NR | NR |
| AC_CUP | 86.8% | 4.7% | 34.0% | 58.5% | 45.8% |
| AC_MOX_CUP | 29.1% | NR | NR | NR | NR |
| AC_WN | 71.0% | NR | 48.7% | 57.3% | NR |
| FN | 53.3% | NR | NR | NR | 25.6% |
| MOX | 15.0% | 58.6% | 51.6% | 79.0% | 90.5% |
| MOX_AAT | 40.0% | NR | 34.4% | 37.6% | 34.0% |
| MOX_AC | 72.2% | NR | 35.4% | 92.6% | 36.9% |
| MOX_WN | 89.1% | NR | NR | NR | NR |
| TCM_WN | 68.0% | NR | NR | NR | 61.5% |
| UT | 0.2% | 66.2% | 27.4% | 32.0%% | 13.5% |
| WN | 30.4% | 55.4% | 47.95% | 56.1% | 37.3% |
| AI | NR | NR | 95.8% | 10.2% | 99.4% |
|  |  |  |  |  |  |

NR: NOT REPORT

MOX: moxibustion; AC: acupuncture; TCM: traditional Chinese medicine; AAT point application; UDD: ultrasonic drug penetration; FN: floating needle; WN: warm acupuncture and moxibustion; AI: point injection; CUP: cupping.

F1: curative effect; F2: IL-2; F3: TNF-a, F12, IL-6; F13: CRP


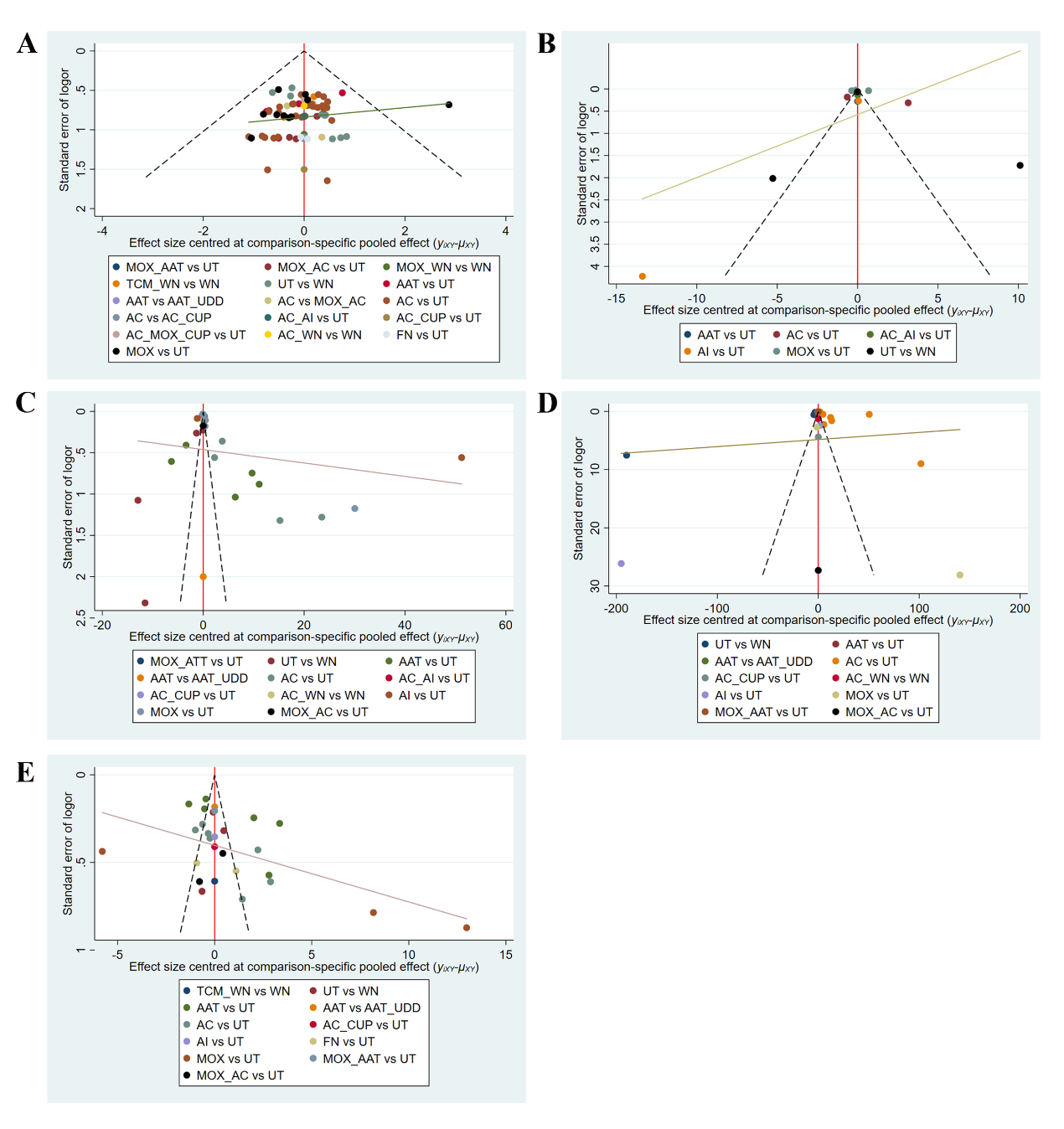


**Supplementary Figure 1** Funnel plots. (A): Efficacy; (B): IL-2; (C): TNF-α; (D): IL-6; (E): CRP.
